# Bioorthogonal Postlabeling Reveals Nuclear Localization of a Highly Cytotoxic Half‐Sandwich Ir(III) Tetrazine Complex in Live Cells

**DOI:** 10.1002/cbic.202500090

**Published:** 2025-04-14

**Authors:** Alfonso Annunziata, Sadek Amhaz, Jérémy Forté, Geoffrey Gontard, Romain Morichon, Joëlle Sobczak‐Thépot, Michèle Salmain

**Affiliations:** ^1^ Institut Parisien de Chimie Moléculaire (IPCM) Sorbonne Université, CNRS F‐75005 Paris France; ^2^ Nanosciencia Faraday 9 28049 Madrid Spain; ^3^ Centre de Recherche Saint Antoine (CRSA) INSERM, Sorbonne Université F‐75012 Paris France; ^4^ Chimie Physique et Chimie du Vivant (CPCV), ENS, CNRS PSL University Sorbonne Université F‐75005 Paris France

**Keywords:** antitumor agents, cellular imaging, fluorescent probes, inverse electron‐demand Diels–Alder, iridium, solvolysis, strained alkynes

## Abstract

Intracellular imaging of anticancer metallodrugs often relies on prelabeling with organic fluorophores, which significantly affects their physicochemical properties and intracellular distribution. On the other hand, the reported postlabeling strategies based on click‐chemistry reactions require cell fixation and permeabilization. Here, this study presents a postlabeling approach based on the catalyst‐free, inverse electron‐demand Diels–Alder reaction (iEDDA) between a strained fluorescein‐tagged bicyclononyne derivative (**BCN‐FAM**) and half‐sandwich Ir(III) complexes containing bidentate ligands comprising a tetrazine (**Tz‐R,R’**) entity. Five half‐sandwich Ir(III) complexes with formula [Cp*Ir(**Tz‐R,R’**)Cl]^0/+^ have been synthesized and fully characterized, including the X‐ray crystal structures of three of the five derivatives. Investigations of their stability and their reactivity in aqueous solution and in a model iEDDA reaction reveal the strong influence of the tetrazine ligand structure on the chemical properties of the corresponding complexes. A highly cytotoxic metallodrug candidate (**Ir‐C,N**_**Ph,Me**_) is identified from biological studies, and chemical reactivity studies disclose an unusual preference for binding of methionine over cysteine. *Postlabeling* of **Ir‐C,N**_**Ph,Me**_ in *live* HeLa cells highlights its preferential accumulation within the nucleus, suggesting its retention through covalent modifications of nuclear proteins in good agreement with other half‐sandwich iridium(III) complexes.

## Graphical abstract

This study reports the synthesis of five tetrazine‐coordinated, half‐sandwich iridium complexes and extensively describe their reactivity. Live cell fluorescent bioorthogonal labeling reveals the nuclear tropism of the most cytotoxic complex.

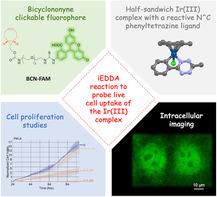

## 1 Introduction

The remarkable success of cisplatin as an anticancer drug^[^ ^1^ ^]^ has paved the way to explore other transition metal complexes for therapeutic purposes.^[^ ^2^ ^]^ Over the last decades, novel complexes of Pt,^[^ ^3^, ^4^, ^5^, ^6^ ^]^ Ru,^[^ ^7^, ^8^ ^]^ Au,^[^ ^9^, ^10^ ^]^ Ir,^[^ ^11^, ^12^ ^]^ and Os^[^ ^13^, ^14^ ^]^ have been proposed as anticancer metallodrug candidates. Among them, half‐sandwich iridium(III) complexes of general formula [(η^5^‐Cp^R^)Ir(X‐Y)Z], (Cp^R^ = cyclopentadienyl derivative, X–Y = bidentate ligand, and Z = monodentate ligand) showed remarkable antiproliferative activity on cell cultures^[^ ^15^, ^16^, ^17^, ^18^ ^]^ and mouse tumor models.^[^ ^19^ ^]^ The nature of the X–Y and the Cp^R^ ligands in this structure has been found to drive both the chemical reactivity and the biological activity of the resulting compounds.^[^ ^20^, ^21^ ^]^ Indeed, depending on the nature of the X–Y ligand, the corresponding complexes were found to target different cellular organelles and interact with different biological targets, as DNA,^[^ ^22^ ^]^ proteins,^[^ ^23^ ^]^ and redox active biomolecules.^[^ ^24^ ^]^ In general, studying the chemical reactivity in physiological condition, uncovering the intracellular localization, and identifying the possible biochemical targets are key aspects to be disclosed when a new metallodrug candidate is designed.^[^ ^25^ ^]^

**Figure 1:**
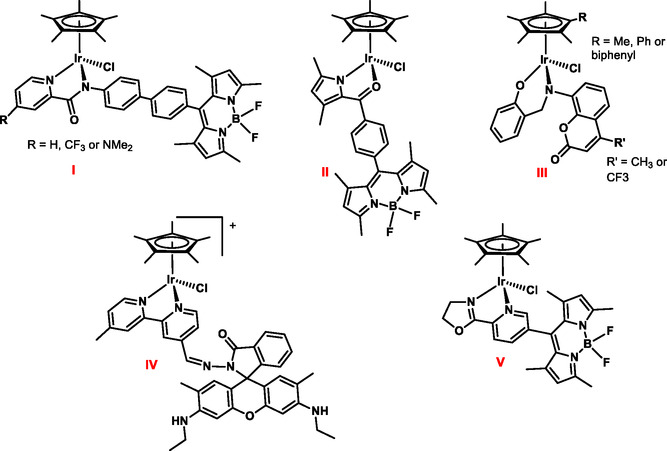
Representative examples of half‐sandwich Ir(III) complexes conjugated to organic fluorophores (BODIPY for I, II, and V; coumarin for III; and rhodamine for IV).

The most popular strategy to track a nonfluorescent metal complex inside cells involves its conjugation to an organic fluorophore.^[^ ^26^ ^]^ Some representative examples of fluorescent half‐sandwich Ir(III) conjugates are reported in **Figure** **1**.

Do and coworkers reported three half‐sandwich iridium complexes with picolinamide‐BODIPY‐conjugate ligands (I in Figure 1) for in‐cell transfer hydrogenation.^[^ ^27^ ^]^ The three derivatives distribute in mitochondria and lysosomes. Similarly, preferential mitochondrial accumulation was observed for Ir(N,*O*‐BODIPY) complexes (II in Figure 1).^[^ ^28^ ^]^ Noteworthy, the same distribution was observed for (η^6^‐*p*‐cymene)Ru(II) and Cp*Rh(III) derivatives bearing a BODIPY‐tagged ligand. Coumarin and rhodamine were also used for conjugation, as in Cp^R^ coumarin‐salicylaldehyde Schiff base containing compounds^[^ ^29^ ^]^ (III in Figure 1) and a rhodamine‐conjugated bipyridine complex^[^ ^30^ ^]^ (IV in Figure 1). Complexes III and IV were found to preferentially accumulate in lysosomes. Most noticeably, the free ligand of complex IV localized in the same cellular compartment, suggesting that the fluorophore itself governed the intracellular localization of the conjugate metallodrug. This trend was also observed with a BODIPY‐conjugate 2‐phenyl‐oxazoline iridium complex^[^ ^31^ ^]^ (V in Figure 1) that was found to accumulate in the endoplasmic reticulum and in mitochondria and then, due to the hydrophobicity of the BODIPY, to be trapped in lipid droplets and delivered to other cell compartments. These examples evidence how fluorescent reporters, usually highly extended aromatic hydrophobic fragments, markedly influence and in some cases entirely govern the intracellular distribution of anticancer metallodrugs.^[^ ^32^, ^33^ ^]^ Therefore, alternative strategies to track metal‐based drugs inside the cell would be highly beneficial to have a more comprehensive and unbiased picture of their intracellular distribution.

Bioorthogonal chemistry has become an essential tool to study biological systems.^[^ ^34^ ^]^ The concepts of bioorthogonal chemistry have been successfully applied to metal complexes.^[^ ^35^ ^]^ Many metal derivatives decorated with alkyne or azide fragments ready for copper‐catalyzed azide‐alkyne cycloaddition (CuAAC) have been reported.^[^ ^36^ ^]^ However, the well‐known toxicity of Cu(I) catalyst^[^ ^37^ ^]^ restricts postlabeling by CuAAC to fixed cells.^[^ ^38^, ^39^ ^]^ One of the most powerful bio‐orthogonal reactions is the [4 + 2]‐cycloaddition of 1,2,4,5‐tetrazines (**Tz‐R,R’**) or 1,2,4‐triazines with dienophiles, known as inverse electron demand Diels–Alder (iEDDA) reaction.^[^ ^40^ ^]^ Due to its high reaction rates in catalyst‐free conditions, high selectivity, and biocompatibility, iEDDA has been successfully applied in cell imaging,^[^ ^41^, ^42^, ^43^, ^44^, ^45^ ^]^ as well as for selective delivery of drugs and radiopharmaceuticals.^[^ ^46^, ^47^, ^48^ ^]^ s‐Tetrazines make useful ligands for a variety of transition metals^[^ ^49^, ^50^ ^]^ and have been used as well as 1,2,4‐triazines in combination with organometallic scaffolds for biological applications. Lo and coworkers designed octahedral bis‐cyclometallated luminogenic Ru, Ir, and Re complexes incorporating a tetrazine entity acting as a quencher.^[^ ^51^, ^52^, ^53^, ^54^ ^]^ Upon iEDDA reaction with strained alkenes and alkynes in living cells, emission is restored, making these complexes useful imaging probes and photosensitizers. Similarly, Kozhevnikov et al. used triazines as ligands in cyclometallated iridium complexes to produce luminescent antibody bioconjugates.^[^ ^55^, ^56^ ^]^

**Scheme 1:**
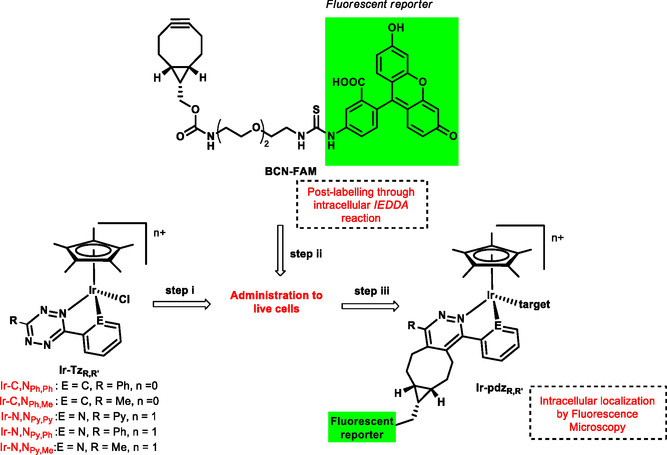
Postlabeling strategy developed in this work.

As a part of our research on half‐sandwich Ir(III) anticancer metallodrugs, we recently combined CuAAC to cryo‐XRF to identify the mitochondria and the actin cytoskeleton as key targets of a complex bearing a 2‐phenyl‐oxazoline decorated with an azide functionality.^[^ ^57^ ^]^ In the present study, we intend to exploit the reactivity of tetrazine in iEDDA reactions by including it in bidentate ligands (X–Y) of half‐sandwich Ir(III) complexes (**Ir‐Tz**_**R,R’**_ in **Scheme** **1**) and propose a postlabeling strategy to localize them in *live cells*. This approach involves the incubation of the nonluminescent iridium complex (Step i) and subsequent iEDDA reaction (Step ii) with a fluorescein‐conjugate derivative (**BCN‐FAM)** of **BCN‐OH** ((1R,8S,9s)‐bicyclo[6.1.0]non‐4‐yn‐9‐ylmethanol)), to finally localize the fluorescein‐tagged complex through confocal microscopy (Step iii).

In detail, we synthesized and characterized five novel half‐sandwich Ir(III) complexes bearing N‐N’ pyridyl‐ (**Tz‐Py,R**) or N‐C^−^ phenyltetrazines (**Tz‐Ph,R**) as X‐Y chelating ligands. We studied their chemical reactivity in coordinating solvents and aqueous solution, and then in iEDDA reaction, as well as their biological properties. Collected results are discussed to rationalize how the different tetrazine ligands affect the chemical reactivity and biological properties of the complexes. Fluorescent postlabeling by iEDDA reaction of the highly cytotoxic derivative **Ir‐C,N**_**Ph,Me**_ allowed to visualize and localize it in both *live* and fixed HeLa cells.

## 2 Results and Discussion

### 2.1 Synthesis and Characterization

**Scheme 2:**
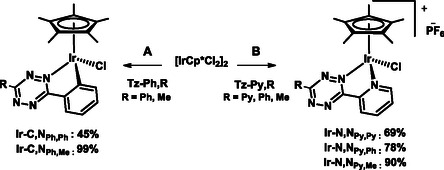
Synthetic routes to the Ir(III) tetrazine complexes. Procedure A: (i) AcONa (3 eq.), MeOH, RT, 15 min; (ii) **Tz‐Ph,R**, RT, 24–72 h. Procedure B: (i) **Tz‐Py,R**, MeOH, RT, 24 h; (ii) NH_4_PF_6_ (3 eq.).

The iridium complexes were synthesized according to the procedures reported in **Scheme** **2**.

**Figure 2:**
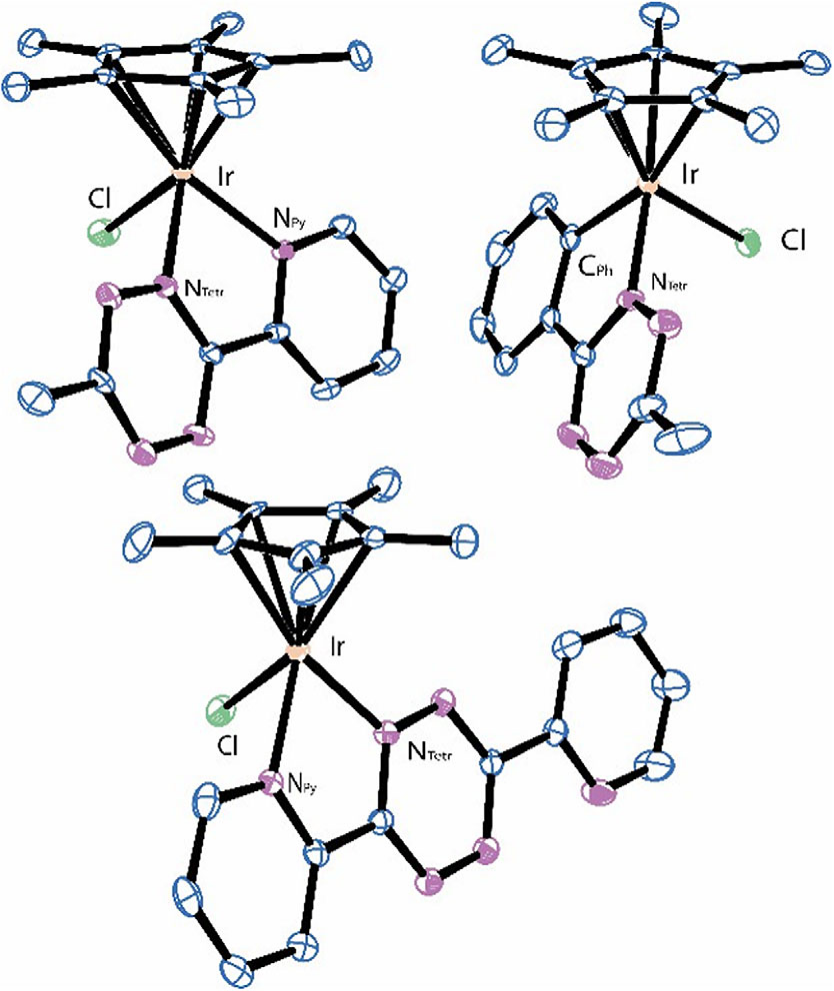
Molecular structures of **Ir‐N,N**_**Py,Me**_ (top left), **Ir‐C,N**_**Ph,Me**_ (top right), and **Ir‐N,N**_**Py,Py**_ (bottom). Hydrogen atoms and counterions were omitted for clarity.

The cationic pyridyl compounds **Ir‐N,N**_**Py,R**_ were isolated as hexafluorophosphate salts by reacting the dimeric precursor [IrCp*Cl_2_]_2_ with the appropriate ligand **Tz‐Py,R** and subsequent addition of ammonium hexafluorophosphate. The neutral derivatives **Ir‐C,N**_**Ph,R**_ were synthesized via C–H metalation promoted by sodium acetate in methanol. All the products were characterized by mono‐ and bidimensional NMR techniques (Figure S1–S5, Supporting Information). In the ^1^H NMR spectrum of **Ir‐N,N**_**Py,R**_, complexes, a downfield shift of the aromatic protons was observed with respect to the free tetrazine ligands **Tz‐Py,R**. The inequivalence of the two pyridyl substituents (one coordinated to the metal and the other not) in the spectrum of **Ir‐N,N**_**Py,Py**_ confirmed the identity of the mononuclear iridium complex (see note). The occurrence of cyclometallation in **Ir‐C,N**_**Ph,R**_ was evidenced by ^1^H‐NMR, with the presence of four inequivalent C—H phenyl protons, as expected for an orthocyclometallated product.^[^ ^58^ ^]^ Also in this case, for the symmetrically substituted **Tz‐Ph,Ph,** two distinct resonance patterns for the non‐equivalent phenyl groups were observed in the ^1^H‐ and ^13^C‐NMR spectra, respectively, confirming the formation of the mononuclear iridium species. The methyl protons in the Cp* ligands appeared as singlets in the range 1.7–1.9 ppm, while the aromatic *C*‐Me carbons of Cp* resonated as singlets between 90 and 95 ppm in ^13^C‐NMR spectra. The identity and the purity of the compounds were further unequivocally assessed by electrospray ionization‐high‐resolution mass spectrometry (ESI‐HRMS), and by RP‐HPLC (Figure S7–S11, Supporting Information). Moreover, the crystal structures of **Ir‐C,N**_**Ph,Me**_, **Ir‐N,N**_**Py,Me**_, and **Ir‐N,N**_**Py,Py**_ were solved by X‐ray diffraction analysis (**Figure** **2**).

All three compounds display the expected pseudo‐octahedral “piano‐stool” geometry with the iridium *η* ^*5*^ *‐*bound to the pentamethylcyclopentadienyl ligand. The chelating tetrazine and the chlorido ligands occupy the “leg positions” in the coordination sphere. Selected bond distances and angles (Table S3 and S4, Supporting Information) agree with those reported for other half‐sandwich Ir(III) derivatives.^[^ ^20^ ^]^ Structural comparison between the analogous methyl‐tetrazine derivatives **Ir‐N,N**_**Py,Me**_ and **Ir‐C,N**_**Ph,Me**_ revealed a shorter Ir‐centroid distance for the pyridyl complex (1.793 *vs.* 1.831 Å), ascribable to its cationic nature that results in a tighter binding of the anionic cyclopentadienyl ligand. No significative difference was observed in the Ir—Cl bond lengths for the three compounds, while a shorter Ir‐N(Tz) distance was observed for **Ir‐C,N**_**Ph,Me**_ with respect to its cationic congener (2.024(7) vs. 2.058(4) Å). A significantly different angle *N(Tz)‐Ir‐Cl* was also found (87.13° vs. 78.59° for **Ir‐N,N**_**Py,Me**_ and **Ir‐C,N**_**Ph,Me**_, respectively). Comparison between cationic **Ir‐N,N**_**Py,Me**_ and **Ir‐N,N**_**Py,Py**_ revealed no significative differences.

### 2.2 In‐Solution Ir‐Cl Cleavage

The cleavage of the Ir—Cl bond is generally believed to be the activation step of half‐sandwich iridium complexes in biological systems, as the formed aqua‐species (Ir—OH_2_) are highly reactive toward biological nucleophiles.^[^ ^59^ ^]^ However, the extent of hydrolysis and the chemical properties of the resulting solvento species are strictly dependent on the nature of the chelating ligand.^[^ ^60^ ^]^ Therefore, we investigated the cleavage of the Ir—Cl bond for our tetrazine complexes in coordinating organic solvents and in aqueous solutions to evaluate the effect of the tetrazine ligand on solvolysis.

### 2.3 Solvolysis in Organic Solvents

We first investigated the stability of the complexes in pure acetonitrile (MeCN‐d_3_) or dimethylsulfoxide (DMSO‐d_6_), by ^1^H‐NMR. These solvents were chosen as MeCN was used in iEDDA reactivity studies and DMSO as vehicle for biological experiments. The same single set of peaks was observed in the ^1^H NMR spectrum of the iridium complexes 5 min after dissolution and after 24 h in both MeCN‐d_3_ or DMSO‐d_6_, except for **Ir‐N,N**_**Py,Py**_ that showed two sets of peaks with a ratio 82:18 after 24 h in DMSO‐d_6_. Addition of 1 equiv. AgBF_4_ to the solutions of the complexes in MeCN‐d_3_ or DMSO‐d_6_ after 24 h resulted in a general downfield shift of the proton peaks, consistent with the formation of solvento species (see, e.g., the spectra of **Ir‐C,N**_**Ph,Me**_ in Figure S12–S13, Supporting Information). This indicated that the complexes (except **Ir‐N,N**_**Py,Py**_ in DMSO‐d_6_) had not spontaneously undergone Ir—Cl cleavage in DMSO or MeCN in absence of silver salt. The second set of peaks in the ^1^H NMR spectrum of **Ir‐N,N**_**Py,Py**_ in DMSO‐d_6_ was then attributed to the Ir‐DMSO adduct (18% conversion). The lack of reactivity, ascribed to the electronic properties of the tetrazine ligand, is notably different from the vast majority of reported half‐sandwich Ir(III) metallodrugs that undergo rapid ligand exchange in DMSO,^[^ ^61^, ^62^ ^]^ due to the high affinity of iridium complexes toward DMSO.^[^ ^63^ ^]^

### 2.4 Solvolysis in Aqueous Solution

Aquation was studied for four of the five complexes by ^1^H‐NMR spectroscopy in acetone‐d_6_–D_2_O mixture (**Ir‐C,N**_**Ph,Ph**_ was disregarded because it precipitates from the solution even with a small percentage of water). Conversions to the corresponding aquo‐species are reported in **Table** **1**, column I.

**Table 1:** Conversion of the Ir‐complexes ([Ir] = 5 mm) to the solvento species in different conditions measured by integration of ^1^H NMR peaks; conversion was measured after 24 h at room temperature.

	**I**	**II**	**III**
**Solvent mix**	Acetone‐d_6_/D_2_Oa)	DMSO‐d_6_/D_2_Oa)	Acetone‐d_6_/D_2_O **(150 m** m **NaCl) **+ 10% DMSO‐d_6_
**Complex**	% Ir‐D_2_O	% Ir‐DMSO	% Ir‐DMSO
**Ir‐C,N**_**Ph,Me**_	11%	67%b)	32% **(15%)** d)
**Ir‐N,N**_**Py,Py**_	>95%	>95%	>95% **(** **>** **95%)**
**Ir‐N,N**_**Py,Ph**_	30%	41%	38% **(23%)**
**Ir‐N,N**_**Py,Me**_	<5%	37%	24% **(17%)**
	–	76%c)	–

a) 1:1 mixture.

b) acetone‐d_6_/D_2_O/DMSO‐d_6_ 4:3:3.

c) D_2_O/DMSO‐d_6_ 4:1.

d) presence of 150 mm NaCl.

While, the neutral compound **Ir‐C,N**_**Ph,Me**_ underwent partial aquation with a conversion of 11% after 24 h, almost no aquation of the cationic counterpart **Ir‐N,N**_**Py,Me**_ was detected over several days, even when dissolved in acetone‐d_6_/D_2_O 1:4 mixture. Thus, electronic factor related to the N‐N/N‐C^−^ ligands seem to affect the Ir—Cl bond strength. The two other pyridyl complexes, **Ir‐N,N**_**Py,Ph**_, and **Ir‐N,N**_**Py,Py**_ underwent partial to total aquation. Thus, the extent of aquation is greatly influenced by substitution patterns on the tetrazine ligand and appears to be related to the electron‐withdrawing strength of the R substituent in **Tz‐Py,R** ligands (Me < Ph < Py).

We next studied the Ir—Cl bond cleavage in DMSO‐d_6_–D_2_O mixture, and results are reported in Table 1, column II. Interestingly, the Ir‐DMSO and not Ir—OH_2_ adducts were formed for all the complexes (Figure S14–S15, Supporting Information). Complete conversion to the DMSO‐species was slowly reached, as **Ir‐C,N**_**Ph,Me**_ gave rise to 67% conversion after 24 h and >95% after 48 h. In the **Ir‐N,N**_**Py,R**_ complexes series, conversion to the DMSO adducts followed the same trend as that observed above for the formation of the aquo‐complexes, with complete conversion observed over several days and rate of conversion following the trend Me ≈Ph << Py. Increased conversion to the DMSO adduct of **Ir‐N,N**_**Py,Me**_ (76% vs. 37%) was observed in DMSO‐d_6_‐D_2_O (1:4) after 24 h. In a nutshell, water appears necessary to trigger the cleavage of the Ir—Cl bond, possibly owing to the increased polarity of the medium that favors the ionization. These results also suggest that the aquo‐complexes are transient intermediates, as similarly observed in Os(II) half‐sandwich metallodrugs.^[^ ^64^ ^]^

As cell culture media contain a high concentration of chloride (≈150 mm),^[^ ^20^ ^]^ we finally studied the effect of chloride concentration on the formation of the DMSO adducts. To this purpose, the ^1^H NMR spectra of the complexes were collected in acetone‐d_6_/D_2_O containing 10% DMSO (acetone was necessary for solubility reasons) or in the same medium supplemented with 150 mM NaCl. As shown in Table 1, column III, almost quantitative conversion of **Ir‐N,N**_**Py,Py**_ to its DMSO adduct still occurred, confirming once again that the pyridyl substituent made the Ir—Cl bond more labile, while the conversion to the Ir‐DMSO adducts was reduced by half for the three other complexes.

### 2.5 iEDDA Reactivity

#### 2.5.1 Kinetic Constant Measurement

To understand how the coordination environment of the newly synthesized half‐sandwich complexes influences the reactivity of the tetrazine ligand in iEDDA, we measured the second order kinetic constants (k_2_) of the reaction with **BCN‐OH** as a model dienophile in MeCN, under pseudo‐first‐order conditions by ultraviolet–visible spectroscopy (Figure S16–S21, Supporting Information).

All the compounds react with **BCN‐OH** to afford the corresponding **Ir‐pdz**_**R,R’**_ complexes where pdz is a pyridazine ligand as confirmed by ESI‐HRMS. Cationic pyridyl complexes **Ir**—**N,N**_**Py,R**_, display very high reaction rates with second‐order rate constants (k_2_, column I in **Table** **2**) between 45.3 ± 0.5 and 286 ± 12 m ^−1^ s^−1^. The accelerating effect follows the trend Me < Ph < Py and is related to the electron‐withdrawing properties of the R substituent, which makes the tetrazine core more electron‐deficient and thus more reactive.^[^ ^65^, ^66^ ^]^ The same trend is observed for the free tetrazines (k_2Tz_ column II in Table 2), measured for comparison. The net positive charge of the **Ir‐N,N**_**Py,R**_ complexes results in a marked accelerating effect of the reaction rate, with *k*_2_/*k*_2Tz_ ratios (column III, Table 2) between 16 and 34, following the R substituent trend Py < Ph < Me.^[^ ^65^, ^66^ ^]^ In terms of frontier molecular orbitals, the major contribution in iEDDA reaction arises from the interaction of the LUMO of the tetrazine with HOMO of the **BCN‐OH** dienophile.^[^ ^67^ ^]^ Coordination of the cationic metal center lowers the energy of the LUMO^[^ ^68^ ^]^ for **Ir‐N,N**_**Py,R**_ complexes, resulting in increased reaction rates, yet this stabilization appears higher for **Ir‐N,N**_**Py,Me**_ than for **Ir‐N,N**_**Py,Ph**_ and **Ir‐N,N**_**Py,Py**_. Distortion energies and intramolecular repulsive interactions also play a key role in the iEDDA reactivity of tetrazines.^[^ ^69^ ^]^ Coordination to the metal scaffold may change the intramolecular motion and interactions of the tetrazine that might be responsible for the higher relative increase in the reaction rate observed for **Ir‐N,N**_**Py,Me**_ compared to the two other derivatives.

**Table 2:** iEDDA second‐order rate constants for complexes and respective tetrazine ligands measured at 24 °C in MeCN.

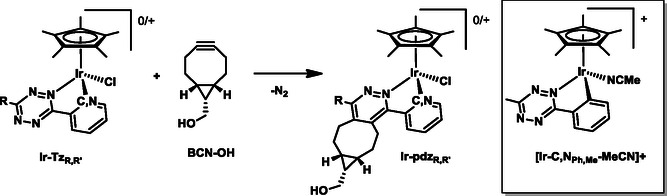
	**I**	**II**	**III**
**Complex**	***k***_**2**_ **(M** ^**−1**^ **.s** ^**−1**^ **)**	**k**_**2Tz**_ **(M** ^**−1**^ **.s** ^**−1**^ **)**	***k***_**2**_ **/** ***k***_**2Tz**_
**Ir‐N,N**_**Py,Py**_	286 ± 12	18 ± 3	16
**Ir‐N,N**_**Py,Ph**_	80 ± 6	4.5 ± 0.2	18
**Ir‐N,N**_**Py,Me**_	45.3 ± 0.5	1.31 ± 0.01	34
**Ir‐C,N**_**Ph,Ph**_	0.732 ± 0.004	1.6 ± 0.2	0.46
**Ir‐C,N**_**Ph,Me**_	0.57 ± 0.01	0.70 ± 0.08	0.81
**[Ir‐C,N**_**Ph,Me**_ **‐MeCN]** ^**+**^	1.8 ± 0.2	0.70 ± 0.08	2.6
**[Ir‐C,N**_**Ph,Me**_ **‐MeCN]** ^**+**^ a)	6.6 ± 0.7	–	–

a) in MeCN/H_2_O 1:1.

Neutral **Ir‐C,N**_**Ph,R**_ complexes display up to 500‐fold lower second‐order rate constants compared to those of **Ir‐N,N**_**Py,R**_. The R substituent trend is similar to that reported above for **Ir‐N,N**_**Py,R**_, with Me < Ph, again ascribable to the electron‐withdrawing effect of the substituent. Moreover, *k_2_/k_2Tz_*  < 1, indicating slower reaction rates for the complexes compared to the uncoordinated ligands. This effect is due to the negatively charged N,C^−^ nature of the ligand, which likely results in the energy increase of the LUMO leading to slower reaction rates. Noteworthy, this effect is more evident in **Ir‐C,N**_**Ph,Ph**_ (*k*_2_/*k*_2Tz_ = 0.46) than in **Ir‐C,N**_**Ph,Me**_ (*k*_2_/*k*_2Tz_  = 0.81), suggesting that the methyl substituent on the tetrazine ring has again a relative beneficial effect on the reactivity of the complex. These results demonstrate that the substitution pattern of the tetrazine core has an impact on the iEDDA reaction rate. However, the charge and the electronic factors remain the major factors contributing to the reactivity of the complexes. To prove it, the cationic acetonitrile derivative **[Ir‐C,N**_**Ph,Me**_ **‐MeCN]** ^**+**^ (see Figure in Table 2) was formed in situ by treating **Ir‐C,N**_**Ph,Me**_ with AgBF_4_ in acetonitrile. The positive charge at the metal is supposed to lower the energy of the LUMO and increase the reaction rate.^[^ ^53^ ^]^ Indeed, a threefold higher rate constant (*k*_2_ = 1.8 m ^−1^ s^−1^) was measured when **[Ir‐C,N**_**Ph,Me**_ **‐MeCN]** ^**+**^ was reacted with **BCN‐OH**. Finally, we performed the reaction between **[Ir‐C,N**_**Ph,Me**_ **‐MeCN]** ^**+**^ and **BCN‐OH** in MeCN/H_2_O 1:1, to validate the use of our complexes in the iEDDA‐based post‐labeling strategy in biological systems. Indeed, the presence of water in the reaction medium was reported to increase the iEDDA reaction rates.^[^ ^70^ ^]^ Gratifyingly, we measured a second‐order rate constant MeCN/H_2_O 1:1 to be *k*_2_ = 6.6 m ^−1^ s^−1^ for **[Ir‐C,N**_**Ph,Me**_ **‐MeCN]** ^**+**^. The collected results evidenced that cationic **Ir‐N,N**_**Py,R**_ complexes are very valid bioorthogonal probe candidates for fluorescence labeling, as the already high k_2_ measured in acetonitrile are expected to be even higher when the reaction is performed in biological systems, where fast reactions are necessary to ensure selectivity in the labeling process.^[^ ^40^ ^]^ However, despite the lower k_2_ values measured in acetonitrile, **Ir‐C,N**_**Ph,R**_ complexes could benefit from the accelerating effect of water along with the formation of cationic adducts in aqueous solution (as described for DMSO in the previous section), making them good candidates for bioorthogonal labeling as well. Overall, the collected data warrant the application of all our half‐sandwich Ir(III) tetrazine complexes for in‐cell labeling.

### 2.6 Biological Studies

The cytotoxic activity of the half‐sandwich Ir(III) compounds was examined in two human cancer cell lines, Huh‐7 (human hepatocellular carcinoma) and HeLa (late‐stage cervix carcinoma), by a label‐free viability assay based on impedance readout, using the real‐time cell analysis (RTCA) system (Figure S22 and S23, Supporting Information).^[^ ^62^, ^71^ ^]^ **Ir‐C,N**_**Ph,Ph**_ was excluded from this study because of its poor solubility in the experimental conditions. Note: according to the speciation studies detailed below, the complexes were administered as their monocationic or dicationic DMSO adducts. IC_50_ values after 72 h incubation were calculated from the proliferation curves and reported in column I of **Table** **3**.

**Table 3:** Partition coefficients, quantities of iridium in HeLa cells after 30 min assayed by ICP‐OES and IC_50_ values measured at 72 h by RTCA (see legend of Figure S26, supporting information).

	I		II	III
**Complex**	**IC**_**50**_ **[μ** m **]**		Iridium quantity [ng/10^6^ cells]	Log P
	**HeLa**	**Huh‐7**		
**Ir‐N,N**_**Py,Me**_	>40	>10	4 ± 0.1a) 17.0 ± 0.4b)	<1
**Ir‐N,N**_**Py,Py**_	>40	>10	N.D.	<1
**Ir‐N,N**_**Py,Ph**_	>40	>10	N.D.	<1
**Ir‐C,N**_**Ph,Me**_	0.20 ± 0.02	0.30 ± 0.01	42.0 ± 0.5c) 578.0 ± 2.3a)	3.7

a) Cells incubated with 2 μm complex.

b) Cells incubated with 20 μm complex; and

c) Cells incubated with 0.2 μm complex.

The IC_50_ values show a contrasting behavior of **Ir‐C,N**_**Ph,Me**_ with respect to the **Ir‐N,N**_**Py,R**_ complexes. Indeed, the three **Ir‐N,N**_**Py,R**_ compounds were found to be inactive up to 10 and 40 μm on the growth of Huh7 and HeLa cells, respectively. Conversely, **Ir‐C,N**_**Ph,Me**_ was highly cytotoxic toward both cell lines, with calculated IC_50_ = 0.20 ± 0.02 and 0.30 ± 0.01 μm on HeLa and Huh7, respectively. Liu and coworkers reported several families of Ir(III) complexes with different X–Y ligands displaying IC_50_ spanning over a wide range of 1–100 μm.^[^ ^18^, ^72^, ^73^, ^74^, ^75^, ^76^ ^]^ 2‐Phenyl‐oxazoline Ir(III) complexes reported by our group display good cytotoxic potencies in the range of 3–6 μm.^[^ ^62^ ^]^ Brabec et al.^[^ ^15^ ^]^ and Pizarro et al.^[^ ^61^ ^]^ reported highly potent half‐sandwich Ir(III) complexes with IC_50_ values from 0.05 to 0.22 μM toward different cancer cell lines. Within this frame, **Ir‐C,N**_**Ph,Me**_ is among the most potent half‐sandwich Ir(III) complexes reported so far.

To better understand the huge difference in the cytotoxic potency displayed by our complexes, we evaluated the lipophilicity of the DMSO adducts by measuring the LogP values (water‐octanol partition coefficient, column II in Table 3). **Ir‐N,N**_**Py,R**_ complexes all display LogP < 1, indicative of a hydrophilic character. Conversely, for complexes **Ir‐C,N**_**Ph,R**_, LogP values of 3.7 and 4.7 were measured for **Ir‐C,N**_**Ph,Me**_ and **Ir‐C,N**_**Ph,Ph**_, respectively. These values indicate a hydrophobic character, logically increasing from the methyl to the phenyl derivative. Such striking difference is expected to affect the cell permeability of the complexes,^[^ ^77^, ^78^ ^]^ which could explain the lack of cytotoxicity observed for **Ir‐N,N**_**Py,R**_ complexes. To confirm this hypothesis, we measured by inductively coupled plasma optical emission spectroscopy (ICP–OES) the content of iridium in HeLa cells treated with **Ir‐C,N**_**Ph,Me**_ and **Ir‐N,N**_**Py,Me**_ for 30 min (Table 3). Taking a mean volume of 3700 μm^3^ for a single HeLa cell, the respective concentration of elemental iridium in cells treated with 2 μm complex was 57 μm for **Ir‐N,N**_**Py,Me**_ and 8280 μm for **Ir‐C,N**_**Ph,Me**_. This latter value illustrates the “sponge‐like” effect^[^ ^79^ ^]^ exerted by the cells toward **Ir‐C,N**_**Ph,Me**_. This excellent cell permeability is probably one of the factors for the high cytotoxic potency of **Ir‐C,N**_**Ph,Me**_ and the lack of cytotoxic activity of **Ir‐N,N**_**Py,Me**_.

### 2.7 Speciation in Biological Medium

Biological studies determined **Ir‐C,N**_**Ph,Me**_ as the best anticancer metallodrug candidate, owing to its high cytotoxicity. To gain further insight into its remarkable potency, we studied by HPLC and ESI‐HRMS its speciation in biological media^[^ ^80^ ^]^ in the presence of DMSO, used as vehicle for cell experiments, and its chemical reactivity with potential biological targets. The nontoxic methyl substituted analog **Ir‐N,N**_**Py,Me**_ was also studied to evaluate whether a different chemical behavior could explain its lack of cytotoxicity.

Speciation of **Ir‐C,N**_**Ph,Me**_ (50 μm) in different media was investigated by RP‐HPLC (Figure S24, Supporting Information). In H_2_O/MeOH 95:5, the corresponding chromatograms recorded at 0 and 3 h showed two peaks at *t*_R_ = 4.1 and 15.6 min, respectively, assigned to the aquo and chlorido complexes. A 68% conversion to the aquo complex was determined from peak areas after 3 h (Table S1, Supporting Information). Addition of 1 mm DMSO (0.007% v/v) gave rise to a mixture of Ir‐DMSO (*t*_R_ = 3.0 min) and Ir‐OH_2_ (*t*_R_ = 4.1 min) adducts in 7/93 ratio after 40 min and the disappearance of the starting chlorido complex (Figure S24B, Supporting Information). Such results support the hypothesis that the aquo species is an intermediate in the DMSO coordination process. Replacement of water by PBS suppressed the hydrolysis (Figure S24C, Supporting Information) and addition of DMSO (0.007% v/v) gave rise to a mixture of the chlorido, aquo, and DMSO complexes in the proportion 75/9/16. Increasing the amount of DMSO up to 0.1% (13 mm) completely shifted the overall equilibrium in favor of the DMSO adduct (Figure S24D, Supporting Information).

**Figure 3:**
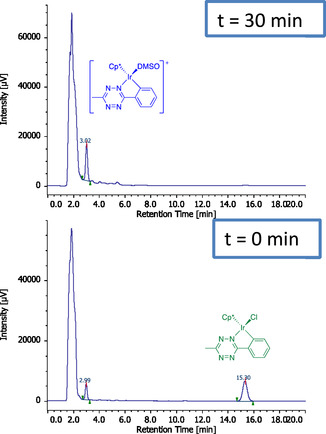
HPLC traces of **Ir‐C,N**_**Ph,Me**_ (10 μm in DMEM + 0.1% DMSO) immediately after dilution and after 30 min at room temperature. The intense peak observed around *t*_R_ = 2 min is due to DMEM components.

Next, the formation of **[Ir‐C,N**_**Ph,Me**_ **‐DMSO]** ^**+**^ was checked in serum‐free cell culture medium (DMEM, **Figure** **3**). Upon dilution to 10 μm of a stock DMSO solution in DMEM to final 0.1% DMSO v/v (Ir/DMSO = 1:1500 mol mol^−1^, i.e., conditions matching those used in cell administration), only the peak at 3.0 min previously assigned to the DMSO adduct was observed within 30 min while the peak at 15.3 min disappeared, in agreement with previous observations in PBS. Conversion of **Ir‐C,N**_**Ph,Me**_ to the DMSO adduct indicates that the starting chlorido complex may not the biologically active species taken up by the cells, and Ir—Cl bond cleavage is the activation step of this metallodrug.^[^ ^81^ ^]^

When this set of experiments was performed with **Ir‐N,N**_**Py,Me**_, only the chlorido complex could be detected by HPLC, while the aqua or DMSO adducts were not retained in any of the tested elution conditions, probably due to their dicationic nature.

We previously observed that other half‐sandwich iridium complexes bind to a wide set of cellular proteins.^[^ ^57^ ^]^ Therefore, to get insight into the possible molecular target(s) of **Ir‐C,N**_**Ph,Me**_, we investigated its reactivity with model amino acids (**AA**), namely N‐acetyl‐L‐histidine (His), N‐Boc‐L‐methionine (Met), and N‐acetyl‐L‐cysteine methyl ester (Cys). The formation of the iridium‐amino acid adducts was first monitored by HPLC after 2 h incubation in H_2_O/MeOH 7:3 at 37 °C. The appearance of new peaks in the chromatogram suggested the formation of covalent adducts (Figure S25, Supporting Information). ESI‐HRMS analysis of the reaction mixtures (Figure S26–S28, Supporting Information) confirmed that cationic adducts of type **[Ir‐C,N**_**Ph,Me**_ **‐AA]** ^**+**^ were formed with His, Cys, and Met by replacement of the chlorido ligand. In contrast, mass spectrometry analysis of mixtures of **Ir‐N,N**_**Py,Me**_ and **AA** showed no binding to Met, while adducts with His and Cys were detected (Figure S29–S30, Supporting Information). At this point, we questioned whether the DMSO adduct reacted similarly with the considered amino acids in DMEM. To investigate this aspect, **[Ir‐C,N**_**Ph,Me**_ **‐DMSO]** ^**+**^ was formed in situ by dilution of a DMSO solution of **Ir‐C,N**_**Ph,Me**_ in DMEM and then incubated with each amino acid at 37 °C for 2 h (Figure S31, Supporting Information). In these conditions, only the sulfur‐containing derivatives Cys and Met were able to displace coordinated DMSO and form **[Ir‐C,N**_**Ph,Me**_ **‐AA]** ^**+**^ adducts, while no His adduct was observed. Furthermore, a competitive binding experiment with an equimolar mixture of the three amino acids for 2 h at 37 °C showed again the preferential formation of adducts with S‐containing amino acids, along with persistence of the DMSO species (Figure S32, Supporting Information). By integration of the peaks areas, the Ir‐Met‐to‐Ir‐Cys ratio was 3:1, suggesting an unusual preferential binding to methionine over cysteine.^[^ ^82^ ^]^ This selectivity may in turn have a strong impact on the biological properties of the complex, by targeting specific cellular protein targets as surface‐accessible, reactive methionines are limited.^[^ ^83^ ^]^ Indeed, S‐donor amino acids are very frequent binding sites for metal complexes in several proteins identified as possible targets for anticancer metallodrugs.^[^ ^84^, ^85^ ^]^ Methionine binding has been involved in the interaction of metal complexes with several proteins^[^ ^25^ ^]^ such as human serum albumin^[^ ^86^ ^]^ and Ctr1, a Cu‐transporter already involved in the cellular uptake of cisplatin.^[^ ^87^ ^]^ On the other hand, the lack of reactivity of **Ir‐N,N**_**Py,Me**_ toward methionine suggests a different set of biomolecular interactions with cellular proteins, and this is another factor, along with the LogP < 1 that could explain its very low cellular uptake and cytotoxicity. Overall, such results provide useful information to guide the identification of the biochemical targets of this tetrazine complex.

Overall, our speciation studies evidence that, upon administration, **[Ir‐C,N**_**Ph,Me**_ **‐DMSO]** ^**+**^ is formed and accumulates inside cells. However, reaction with biological S‐nucleophiles present in the culture medium to form covalent adducts that can also enter the cells by passive diffusion or active transport cannot be ruled out.^[^ ^88^ ^]^

### 2.8 Reactivity with the Bio‐orthogonal Fluorescent Reagent BCN‐FAM

We next selected **Ir‐C,N**_**Ph,Me**_ as candidate for the *postlabeling* iEDDA‐based strategy. Indeed, this highly cytotoxic metallodrug displayed an excellent cell permeability, and we proved that it undergoes Ir‐Cl cleavage to form cationic adducts in aqueous systems. These factors are likely to increase the iEDDA reaction rate, supporting selective and efficient bioorthogonal in‐cell labeling. We synthesized a **BCN‐OH** fluorescein conjugate, **BCN‐FAM** (Scheme 1) as bioorthogonal labeling reagent, following a procedure reported in the literature (Figure S6, Supporting Information).^[^ ^89^ ^]^ Before moving to cell experiments, we tested the iEDDA labeling strategy in vitro.

Reaction between **Ir‐C,N**_**Ph,Me**_ and 2.5 eq. of **BCN‐FAM** in methanol at room temperature afforded the pyridazine product **Ir‐pdz**_**Ph,Me**_ **‐FAM** as a mixture of two diasteroisomers, due to the presence of stereogenic centers on the metal and **BCN‐FAM** with an observed rate constant of 0.017 min^−1^ and a half‐life of 41 min, as monitored by HPLC (Figure S32, Supporting Information). By comparison, **Ir‐N,N**_**Py,Me**_ reacts faster with **BCN‐FAM**, with 54% conversion measured at the first injection (Figure S33, Supporting Information), consistently with the kinetic studies performed on **BCN‐OH**. The identity of the two pyridazine products **Ir‐pdz**_**R,R’**_ **‐FAM** was confirmed by ESI‐HRMS analysis, which showed no other species formed upon reaction. Then, we performed the reaction between **Ir‐C,N**_**Ph,Me**_ and **BCN‐FAM** in cell culture medium (DMEM) containing 1% DMSO. According to the speciation studies, in this condition **[Ir‐C,N**_**Ph,Me**_ **‐DMSO]** ^**+**^ is the only species present in solution. In these conditions, **Ir‐C,N**_**Ph,Me**_ reacts much faster than in MeOH, with 91% of the starting material converted to the pyridazine product at the first injection (<5 min) at room temperature, selectively forming **Ir‐pdz**_**Ph,Me**_ **‐FAM** as a mixture of the two diasteroisomers, as detected by HPLC and HRMS (Figure S34, Supporting Information).

Once we proved the formation of the desired labeled product **Ir‐pdz**_**R,R’**_ **‐FAM,** we measured their fluorescence spectrum in different solvent mixtures (Figure S35, Supporting Information). To this purpose, the reaction between **Ir‐C,N**_**Ph,Me**_ or **Ir‐N,N**_**Py,Me**_, and **BCN‐FAM** was repeated in methanol or acetonitrile using an excess of the iridium complexes to fully consume **BCN‐FAM** and measure only the fluorescence emission of the **Ir‐pdz**_**R,R’**_ **‐FAM** products. Both labeled compounds showed an emission profile similar to that of **BCN‐FAM** with *λ*_em_ in the range of 519–525 nm, with a slight change according to the solvent mixture (methanol, water + 1% DMSO, and DMEM + 1% DMSO were examined). The robust emission of the iEDDA product at 519 nm in DMEM + 1% DMSO, along with the fast and selective ligation in pseudo‐physiological conditions, comforts the translation of the *postlabeling* strategy to *live cells*.

### 2.9 In‐Cell Fluorescence Labeling

**Figure 4:**
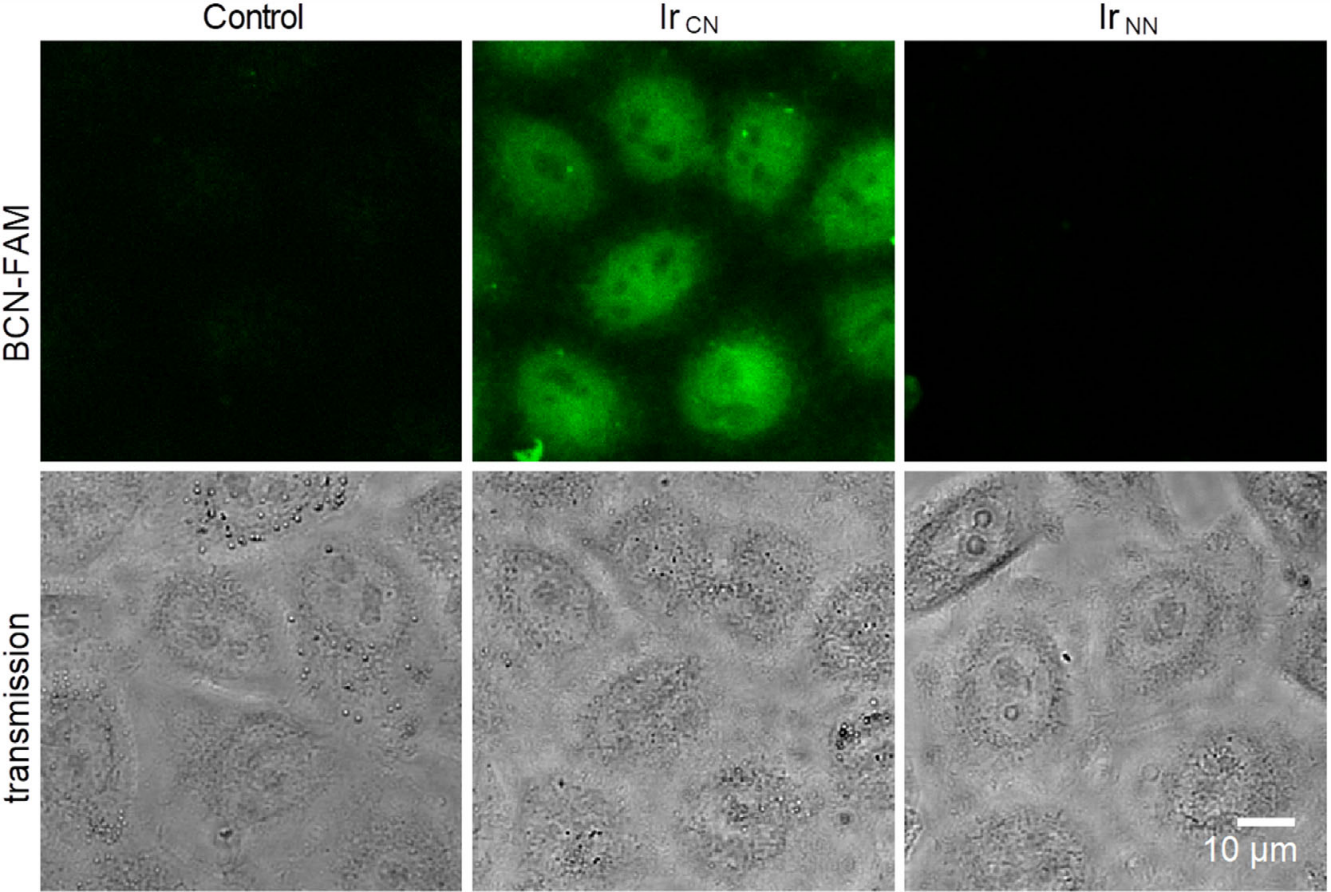
*In vivo* imaging of **Ir‐C,N**_**Ph,Me**_ in HeLa cells using **BCN‐FAM** as a sensor. HeLa cells were sequentially exposed to 2 μm **Ir‐C,N**_**Ph,Me**_ or **Ir‐N,N**_**Py,Me**_ for 30 min, and then to 8 μm **BCN‐FAM** for 4 h. After several washes to remove unbound freely diffusible **BCN‐FAM**, live confocal microscopy was then immediately performed to acquire the fluorescence signal (shown here as the sum of five confocal planes). Cells were also visualized by transmitted light to identify nuclei and nuclear bodies including nucleoli, which appeared darker. A robust fluorescence signal was detected in cells exposed to **Ir‐C,N**_**Ph,Me**_ but not in cells exposed to **Ir‐N,N**_**Py,Me**_ or the DMSO vehicle, indicating the presence of **Ir‐C,N**_**Ph,Me**_ in cells and its tracking with **BCN‐FAM.**

The ability of **Ir‐C,N**_**Ph,Me**_ to undergo iEDDA reaction with **BCN‐FAM** was then examined in live cells. **Ir‐N,N**_**Py,Me**_ was also tested for comparison. HeLa cells were incubated with the complexes for 30 min and then washed with PBS. Then, cells were further incubated for 4 h with medium containing **BCN‐FAM** (8 μm) and washed thoroughly with DMEM prior to confocal microscopy. A minimal concentration of 2 μm of **Ir‐C,N**_**Ph,Me**_ was required to acquire good quality images with **BCN‐FAM,** but no cytotoxic effects were detected during this brief exposure time, while **BCN‐FAM** itself was not toxic up to 40 μm. Interestingly, a strong green fluorescence emission was readily detected in live cells (**Figure** **4**), attributable to the labeled complex **Ir‐pdz**_**Ph,Me**_ **‐FAM.**

Confocal fluorescence images show a robust staining of the complex in the nucleus except darker areas corresponding to nuclear bodies, such as nucleoli. Only a faint staining was detected in the cytoplasm (Figure 4). These results were reproduced in cells that were fixed prior to iEDDA labeling (Figure S36, Supporting Information). This method enhanced the cytoplasmic staining but again showed a significant colocalization of **Ir‐C,N**_**Ph,Me**_ labeled with **BCN‐FAM** and the blue‐fluorescent DNA stain DAPI (Pearson's correlation coefficient 0.62 ± 0.06). No fluorescence signal was detected in cells pretreated with only DMSO vehicle or **Ir‐N,N**_**Py,Me**_ and then incubated with **BCN‐FAM** for 4 h. This confirms that unreacted **BCN‐FAM** is effectively removed by washing, and no unwanted side labeling of biomolecules occurred.^[^ ^90^ ^]^ The absence of fluorescence signal in cells treated with **Ir‐N,N**_**Py,Me**_ is most likely due to its very poor cell uptake. These results proved the efficacy of our *postlabeling* strategy to visualize organometallic complexes in *live cells*.

Preferential accumulation of **Ir‐C,N**_**Ph,Me**_ in the nucleus of cells rather than in other cellular organelles may be tricky to rationalize. Indeed, many factors may affect the subcellular distribution.^[^ ^91^ ^]^ As discussed in the introduction, preferential accumulation of iridium complexes in lysosomes and mitochondria was observed when the metal structures were conjugated to highly hydrophobic fluorescent reporters,^[^ ^27^, ^28^, ^29^, ^30^, ^31^ ^]^ whose physicochemical properties govern the intracellular distribution of the whole conjugate species.^[^ ^32^, ^33^ ^]^ Pizarro and coworkers unambiguously localized a highly cytotoxic cationic pyridine‐tethered iridium complex in mitochondria in MCF‐7 human breast cancer cells, by combining cryo‐SXT and cryo‐XRF tomography techniques.^[^ ^92^ ^]^ Lipophilic cations are known to preferentially accumulate into the mitochondria,^[^ ^93^ ^]^ and in that case the doubly chelate nature of the tethered complex may confer to the complex the appropriate lipophilic‐charge balance to enter this specific organelle. Liu and coworkers reported an half‐sandwich Ir(III) complexes with an imino‐pyridyl ligand^[^ ^94^ ^]^ that also accumulates in the nucleus of A549 lung cancer cells. Notably, the authors reported a LogP value of 3.5^[^ ^94^ ^]^ very close to that of **Ir‐C,N**_**Ph,Me**_. Therefore, the degree of lipophilicity might be considered a factor governing the nuclear accumulation of our complex.^[^ ^91^ ^]^ Li and coworkers reported some cyclometalated iridium complexes of formula [Ir(C‐N)_2_(solv)_2_]^+^ (C‐N = phenylpyridine derivative, solv = DMSO, or MeCN) that preferentially accumulate in the nuclei via protein‐mediated transport of the nuclear pore complexes.^[^ ^95^, ^96^ ^]^ These complexes share with [**Ir‐C,N**_**Ph,Me**_ **‐DMSO]** ^**+**^ the same charge, cyclometalated C–N and DMSO ligands, which could agree with the interaction with proteins involved in NPCs transport. Finally, free diffusion to the nucleus and formation of covalent adducts with nuclear proteins is another mechanism that could explain retention of **Ir‐C,N**_**Ph,Me**_ in the nucleus, as we previously showed for a half‐sandwich iridium complex.^[^ ^57^ ^]^

## 3 Conclusions

To sum up, we have presented a fast and selective in‐cell labeling strategy of Ir(III) half‐sandwich complexes based on the iEDDA reaction between the Ir‐coordinated tetrazine ligand and a fluorescein‐tagged BCN derivative. A library of five complexes featuring a tetrazine‐containing neutral N–N or an anionic N–C^−^ ligand was first produced. Extensive solvolysis studies underscored that the liability of the chlorido ligand was greatly influenced by the nature of the chelating ligand and the solvent composition. Speciation studies in biological medium suggested that the compounds are administered to the cells as their DMSO adducts. Further exchange of the DMSO ligand by S‐containing amino acids was demonstrated with an unusual preference for methionine over cysteine. All the complexes efficiently underwent iEDDA reaction with a model strained alkyne and parameters governing the rate constant value were duly identified, underscoring the possibility to tune the chemical reactivity of the complexes according to the chelating ligand structure. Biological studies revealed a strong cytotoxicity for **Ir‐C,N**_**Ph,Me**_, which was identified as one of the most potent iridium‐based anticancer metallodrugs reported to date. Its high cytotoxicity was correlated to a remarkable level of cellular accumulation. Finally, in vivo labeling of **Ir‐C,N**_**Ph,Me**_ was successfully demonstrated in live HeLa cells, via confocal fluorescence microscopy and revealed a preferential accumulation of the complex in the nucleus.

Our postlabeling strategy provides a valid approach to track cytotoxic metal complexes in *live cells* by fluorescence microscopy, providing qualitative information on their subcellular distribution and highlights the dual functionality of tetrazine as both ligand and bioorthogonal reactive entity. In‐depth biological studies are now required to elucidate the mechanism of action of **Ir‐C,N**_**Ph,Me**_ and establish a precise correlation between its chemical reactivity, its subcellular distribution and its cytotoxicity.

## 4 Notes

The optimized reaction procedure involves addition of a solution of [IrCp*Cl_2_]_2_ to a solution containing the tetrazine ligand. Addition of ligand **Tz‐Py,Py** to the solution of Ir dimer resulted in the formation of a side product beyond complex **Ir‐N,N**_**Py,Py**_. Attempts to separate the two compounds led to isolate crystals that were identified as a bimetallic species according to its X‐Ray diffraction analysis (Figure S37, Supporting Information) that was not characterized any further.

## Conflict of Interest

The authors declare no conflict of interest.

## Supporting information

Supplementary Material

## Data Availability

Deposition Numbers 2324970 (for **Ir‐N,N**_**Py,Me**_), 2324971 (for **Ir‐C,N**_**Py,Me**_), 2324972 (for **Ir‐N,N**_**Py,Py**_), and 2324973 (for **Ir**_**2**_ **(N,N**_**Py,Py**_ **)(Cl)**_**2**_) contain the supplementary crystallographic data for this paper. These data are provided free of charge by the joint Cambridge Crystallographic Data Centre and Fachinformationszentrum Karlsruhe. The data that support the findings of this study are available in the supplementary material of this article.
