# Supplementary material for: Bioorthogonal Postlabeling Reveals Nuclear Localization of a Highly Cytotoxic Half‐Sandwich Ir(III) Tetrazine Complex in Live Cells
Source: Chembiochem. 2025 Apr 14;26(12):e202500090. doi: 10.1002/cbic.202500090 (PMC12177701; doi:10.1002/cbic.202500090)
Supplement: Supplementary file 1 — Supplementary Material [file CBIC-26-e202500090-s001.pdf]

Bioorthogonal Post-Labeling Reveals Nuclear Localization of a Highly Cytotoxic Half-sandwich Ir(III) Tetrazine Complex in Live Cells

Supplementary Information

Alfonso Annunziata, Sadek Amhaz, Jérémy Forté, Geoffrey Gontard, Romain Morichon, Joëlle Sobczak-Thépot, and Michèle Salmain

Content

MATERIALS **2**

INSTRUMENTATION **2**

ANALYTICAL EXPERIMENTS **3**

Ir-Cl bond cleavage by NMR 3
Kinetic measurements of IEDDA reaction 3
Partition coefficient (log P) determination 3
ICP-OES analysis 3
Speciation and amino acids binding by HPLC 4
Amino acids binding by HRMS-ESI-MS 4
Cell culture and real time cell analysis (RTCA) 4
Fluorescence Microscopy 4

SYNTHESIS AND CHARACTERIZATION **5**

General procedure for the synthesis of Ir-N,$N_{Py,R}$ 5
General procedure for the synthesis of Ir-C,$N_{Ph,R}$ 5
Synthesis of BCN-FAM 6
References 6

SUPPLEMENTARY FIGURES **7**

NMR spectra 7
In-solution NMR studies 15
iEDDA Reactivity with BCN-OH 18
Reaction with amino acids 25

IN-CELL IEDDA POST-LABELLING **34**

CRYSTALLOGRAPHIC DATA AND TABLES **35**

Materials

$[IrCp^*Cl_2]_2$ and 3,6-diphenyl-1,2,4,5-tetrazine (**Tz-Ph,Ph**) were purchased from TCI. 3,6-di-2-pyridyl-1,2,4,5-tetrazine (**Tz-Py,Py**) and N-Acetyl-L-histidine (His) were purchased from Fluorochem. Ammonium hexafluorophosphate, N-Boc methionine, N-Boc-L-methionine (Met), and N-Acetyl-L-cysteine (Cys), (1R,8S,9s)-Bicyclo[6.1.0]non-4-yn-9-ylmethanol (**BCN-OH**), N-[(1R,8S,9s)-Bicyclo[6.1.0]non-4-yn-9-ylmethyloxycarbonyl]-1,8-diamino-3,6-dioxaoctane (BCN-$(EG)_2$-NH_2) and fluorescein isothiocyanate (**FITC**) used to synthesize **BCN-FAM** were obtained from Sigma-Aldrich. DCM, MeOH, MeCN, DMSO were used without further purification. Deuterated solvents were obtained from Eurisotop. Gibco Minimum Essential Medium (MEM) for cell culture and Dulbecco's Modified Eagle Medium (DMEM) for both cell-free experiments and cell culture were obtained from Thermo-Fisher, as were all cell culture supplements. Non-symmetrical tetrazine ligands (**Tz-Ph,Me**, **Tz-Py,Me** and **Tz-Py,Ph**) were synthesized according to literature procedures.[1,2]

Instrumentation

NMR spectroscopy. ^1H- and ^{13}C-NMR, mono- and bi-dimensional spectra were recorded in 5 mm NMR tubes using a Bruker Nanobay 300 MHz or a Bruker Nanobay 400 MHz spectrometers at room temperature. ^1H and ^{13}C NMR chemical shifts were referenced to solvent peaks residues: DCM-d_2 (5.3 and 53.52 ppm), $CDCl_3$ (7.26 ppm), Acetone-d_6 (2.09 and 30.60 ppm), MeCN-d_3 (1.96 ppm), DMSO-d_6 (2.52 ppm) D_2O (4.79 ppm). Data analysis was performed using MestreNova, version 6.0 (Mestrelab Research, S.L.).

UV-Vis spectroscopy. UV-vis absorption spectra were recorded on a Cary 50 spectrophotometer (Varian) in 1-cm pathlength quartz cuvettes (800 µL – 3 mL). Kinetic curves were analyzed using Varian software

Fluorescence spectroscopy. Fluorescence spectra were collected using a FP-6200 spectrofluorimeter (Jasco) in 1-cm path length quartz cuvettes (800 µL). Spectra were recorded at 125 nm/min scan rate, 1 nm data pitch and 5 nm bandwidth.

HPLC analysis. HPLC analysis was performed using a HPLC system (Jasco) comprising PU-2080 and PU-2087 pumps coupled to a uv-visible detector (UV-2075) set to 254 nm. Separations were performed on a 4.6 x 150 mm Nucleodur C18 HTec 5 µm column (Macherey-Nagel). Elution conditions are summarized in the following table. Eluents used in conditions 1-x were 0.1% TFA, 0.9 % NaCl in water (A) and 0.1% TFA in MeCN (B) at 0.8 ml/min.

Condition	eluent
1-1	5% B
1-2	45% B
1-3	55% B
1-4	70% B
1-5	0-5min 35% B, 5-15 min 35-55% B, 15-20 min 55% B, 20-25 min 55-75% B, 25-30 min, 75-35% B, 30-35 min 35% B

X-ray crystallography. Single crystals were selected, mounted onto a cryoloop and transferred into a cold nitrogen gas stream. Intensity data were collected with a Bruker Kappa-APEX2 CCD diffractometer using a micro-source CuKα radiation. Data collections, unit-cell parameters determinations, integration and data reductions were performed with the Bruker

APEXIII/SAINT1 suite at 200 K. The structures were solved with SHELXT2 and refined anisotropically by full-matrix least-squares methods with SHELXL3 using Olex24 software (**[Ir-N,N$_{Py,Py}$]PF$_6$** and **[Ir$_2$(N,N$_{Py,Py}$)Cl$_2$](PF$_6$)$_2$**) or WinGX5 package (**[Ir-N,N$_{Py,Me}$]PF$_6$** and **Ir-C,N$_{Py,Me}$**). The structures were deposited at the Cambridge Crystallographic Data Centre with numbers CCDC 2324970-2324973 and can be obtained free of charge via www.ccdc.cam.ac.uk.

ICP-OES analysis. Determination of iridium concentration in cell samples was performed on an ICP-OES 5100 SVDV analyser (Agilent). Iridium calibration standards (8, 16, 31, 63, 125, 250, 500 and 1000 ppb in 2% HNO_3) were prepared using a 10 ppm PlasmaCAL SCP Science® multi-element stock solution. Measurements were performed in triplicate.

Analytical experiments

Ir-Cl bond cleavage by NMR

Acetonitrile and DMSO solutions (10 mM) were prepared by dissolving the Ir-complexes in MeCN-d$_3$ or DMSO-d$_6$ respectively. After preparation of the solutions, ^{1}H-NMR spectra were recorded (the first experiment was collected ca. 5-10 min after dissolution) and then the samples were kept at room temperature and subsequent spectra were recorded at different times over 24 h. After 24 h, an excess of $AgBF_4$ was added to the NMR tube, AgCl was filtered off through a pad of celite and the NMR spectrum of the filtrate solution was recorded. For the experiments in aqueous solutions, the Ir-complexes were dissolved in the appropriate organic solvent (Acetone-d$_6$ or DMSO-d$_6$) and diluted with D_2O or 300 mM NaCl in D_2O to a final concentration of 5 mM. ^{1}H-NMR spectra were recorded (the first spectrum was collected ca. 5-10 min after dissolution) and then the samples were kept at room temperature and subsequent spectra were recorded at different time points over 24 h.

Kinetic measurements of IEDDA reaction

The second order rate constants k_2 for IEDDA reaction between tetrazine ligands (60-200 mM) or the Ir(III)-tetrazine complexes (30-60 mM) in MeCN or MeCN/H_2O (1:1) were measured under pseudo first-order conditions using an excess of the corresponding compound with **BCN-OH** (0.250 – 6 mM) at 298 K by UV spectroscopy. The reactions were monitored by following the increase or the decrease of absorbance at selected wavelengths upon addition of **BCN-OH** to the tetrazine or tetrazine complexes. Data were fitted to a single-exponential equation to give the pseudo-first-order rate constants k_{obs} using Varian software. k_{obs} collected at different excesses was then plotted against the concentration of **BCN-OH** to calculate the second-order rate constant k_2.

Partition coefficient (log P) determination

The partition coefficients (log $P_{o/w}$) of the complexes were determined by an HPLC method according to the Test guideline no. 117 recommended by OEDC, using a series of 7 reference substrates (Benzonitrile, Anisole, Toluene, Naphthalene, Diphenyl ether, n-butylbenzene, Triphenylamine). Elution conditions were 70 % MeCN in 10 mM ammonium acetate in water at a flow rate of 1 ml/min. Solutions of complexes in water / DMSO 99:1 were used for the analysis so that the Log P are measured for the solvento adducts.

ICP-OES analysis

Briefly, 100-mm culture dishes were seeded with 1x10^6 HeLa cells in complete medium. After two days, the medium was removed, and replaced by 10 mL serum-free DMEM supplemented with **Ir-N,N$_{Py,Me}$** (2 or 20 µM) or **Ir-C,N$_{Ph,Me}$** (0.2 or 2 µM). Cells were incubated at 37°C under 5%

CO_2. After 30 min, cell media were carefully collected and centrifuged at 1000 rpm in order to collect the dead floating cells. Supernatants were kept apart and pellets 1 were kept for later use Adherent cells were washed twice with PBS, detached with trypsin, resuspended in serum-free DMEM and added to pellets 1. Suspensions were centrifuged at 1000 rpm to give pellets 2. Supernatants (6.5 mL) were supplemented with HNO_3 68% (0.135 mL) to reach a final concentration of 2%. Pellets 2 were suspended in 0.15 mL HNO_3 68% and sonicated in a sonication bath for 60 min and with a sonication probe for 3x10 s. The volume was completed to 7 mL with water to reach a final HNO_3 concentration of 2%.

Speciation and amino acids binding by HPLC

Stock solution of **Ir-C,N$_{Ph,Me}$** and **Ir-N,N$_{Py,Me}$** (0.75 mM) were prepared in MeOH or DMSO and diluted in H_2O or DMEM to final concentrations of 10-50 µM. HPLC were run at different times.

Amino acids binding by HRMS-ESI-MS

Complexes **Ir-C,N$_{Ph,Me}$** and **Ir-N,N$_{Py,Me}$** were dissolved in H_2O/MeOH 70/30 ([Ir] = 0.25 mM) and the appropriate amino acid derivative (N-acetyl cysteine methyl ester, N-Boc methionine or N-acetyl histidine) was added from stock solutions (6 mM) in water. Reaction mixtures were incubated for 2 h at 37 °C and diluted in MeOH for mass analysis.

Cell culture and real time cell analysis (RTCA)

HeLa (human cervix carcinoma) cells were cultured in DMEM High Glucose. Huh-7 (human hepatoma) cells were cultured in Minimum Essential Medium (MEM) supplemented with 1 % sodium pyruvate and 1 % non-essential amino acids (Gibco, Invitrogen). All media were supplemented with GlutaMAX® (Gibco, Invitrogen®), antibiotics (penicillin, streptomycin) and 10 % fetal bovine serum. All treatments with iridium complexes were performed in medium containing 5% serum. For real time cell analysis, xCELLigence E-plates were calibrated for a baseline definition and cells were seeded at 2,000 cells per well in 100 µL complete medium. After about 24 h, 100 µL of serum free medium containing the iridium complexes were added. Each condition was performed in triplicate and the cell index was measured over a period of 48-96 h (Real Time Cell Analyzer, Agilent). IC_{50} values were calculated from triplicate experiments.

Fluorescence Microscopy

20,000 HeLa cells were seeded into IBIDI 35-mm µ-dishes and cultured overnight in complete medium. Cells were washed with PBS and incubated for 30 min in medium with 5% serum containing either 2 µM **Ir-C,N$_{Ph-Me}$**, **Ir-N,N$_{Pyr,Me}$** or the DMSO vehicle as a control. For live cell microscopy, cells were washed twice with PBS and further cultured for 4h in medium with 5% serum and 8 µM **BCN-FAM**. The cells were then washed three times with DMEM and twice with phenol red-free DMEM, all with 5% serum to remove the non-clicked free diffusible **BCN-FAM**. Live imaging was performed using a FLUOVIEW 3000 confocal microscope with a 100X UPlanSApo ON1.4 oil objective. Alternatively, after culture with the iridium complexes, cells were fixed for 15 min at room temperature with 4 % PFA in PBS. After four PBS washes, the iEDDA reaction was performed with 8 µM **BCN-FAM** in PBS for 4 h at room temperature. Cells were washed twice with PBS and DNA was stained with 300 nM DAPI. Confocal microscopy was performed as described above. Confocal images were processed using Fiji.

Synthesis and Characterization

General procedure for the synthesis of Ir-N,$N_{Py,R}$

$[IrCp^*Cl_2]_2$ (0.12 g, 0.15 mmol) was added to a solution containing the appropriate pyridyl tetrazine ligand (0.36 mmol) in 15 mL of methanol. The mixture immediately turned to dark. After stirring for 24 h at RT, the volume of the mixture was concentrated to ca. 2-3 mL and NH_4PF_6 (0.08 g, 0.5 mmol) was added dissolved in the minimum amount of methanol. The mixture was stored at -20 °C for 24 h during which a dark-brown microcrystalline solid precipitated. This solid was isolated by filtration, washed with small portions of cold methanol and with diethyl ether.

Ir-N,$N_{Py,Py}$. The complex was recrystallized another time with diethyl ether by acetone solution. Yield: 69%. 1H NMR (300 MHz, Acetone) δ 9.34 (d, J = 5.2 Hz, 1H, H2), 9.22 (d, J = 7.5 Hz, 1H, H5), 9.10 (d, J = 4.6 Hz, 1H, H11), 8.92 (d, J = 7.9 Hz, 1H, H14), 8.67 (t, J = 7.5 Hz, 1H, H4), 8.37 – 8.22 (m, 2H, H3 and H13), 7.88 (dd, J = 7.8, 4.7 Hz, 1H, H12), 1.95 (s, 15H). 13C NMR (75 MHz, Acetone) δ 168.31 (C7 or C8), 166.49 (C8 or C7), 154.64 (C2), 153.24 (C14), 150.46 (C6 or C9), 143.17 (C4), 139.70 (C13 or C3), 137.32 (C3 or C13), 133.79 (C9 or C6), 129.52 (C12), 128.86 (C5), 127.33 (C14), 95.35 (x5, Ar-Cp*), 9.43 (x5, Me-Cp*). HRMS (ESI) m/z: $[M-PF_6]^+$ Calcd. for $C_{22}H_{23}ClIrN_6$ 599.1296. Found 599.1277.

Ir-N,$N_{Py,Ph}$. Yield: 78% ^{1}H NMR (300 MHz, Acetone) δ 9.29 (d, J = 5.4 Hz, 1H, H2), 9.14 (d, J = 7.8 Hz, 1H, H5), 8.78 (d, J = 7.7 Hz, 2H, H10 and H14), 8.62 (t, J = 7.8 Hz, 1H, H4), 8.31 – 8.16 (m, 1H, H3), 7.97 – 7.72 (m, 3H, H11, H12 and H13), 1.92 (s, 15H, Me-Cp*). ^{13}C NMR (75 MHz, Acetone) δ 166.02 (x2, C7 and C8), 152.76 (C2), 149.00 (C6), 141.43 (C4), 134.71 (C12), 131.89 (C3), 130.47 (C9), 130.08 (x2, C10 and C14), 129.02 (x2, C11 and C13), 126.82 (C5), 93.45 (x5, Ar-Cp*), 7.92 (x5, Me-Cp*). HRMS (ESI) m/z: $[M-PF_6]^+$ Calcd. for $C_{23}H_{24}ClIrN_5$ 598.1350. Found 598.1344.

Ir-N,$N_{Py,Me.}$ ^{1}H NMR (300 MHz, Acetone) δ 9.27 (d, J = 5.5 Hz, 1H, H2), 9.10 (d, J = 7.9 Hz, 1H, H5), 8.68 – 8.53 (m, 1H, H4), 8.34 – 8.12 (m, 1H, H3), 3.36 (d, J = 25.0 Hz, 3H, H9), 1.86 (s, 15H, Me-Cp*). ^{13}C NMR (75 MHz, Acetone) δ 171.00 (C7 or C8), 158.66 (C8 or C7), 152.75 (C2), 149.00 (C6), 141.43 (C4), 131.89 (C3), 126.71 (C5), 93.26 (x5, Ar-Cp*), 20.85 (C9), 7.69 (x5, Me-Cp*). HRMS (ESI) m/z: $[M-PF_6]^+$ Calcd. for $C_{18}H_{22}ClIrN_5$ 536.1188. Found 536.1176.

General procedure for the synthesis of Ir-C,$N_{Ph,R}$

$[IrCp^*Cl_2]_2$ (0.12 g, 0.15 mmol) and sodium acetate (0.037 g, 0.45 mmol) were stirred in 10 mL of methanol for 10 min. The appropriate phenyl tetrazine ligand (0.75 mmol) was dissolved in 2 mL dichloromethane and added to the yellow clear solution containing the iridium precursor. The reaction was monitored by TLC and when the iridium starting material disappeared the solvent was evaporated under vacuum. The dark solid residue was extracted with dichloromethane and filtered over a pad of Celite. The products were isolated as dark solids by column chromatography.

Ir-C,$N_{Ph,Ph}$. Reaction time: 5 days at RT. Column chromatography conditions: SiO_2, DCM 100%. Obtained as a black powder, yield: 45%. ^{1}H NMR (300 MHz, CD_2Cl_2) δ 8.63 - 8.55 (m, 2H, H10 and H14), 8.38 (d, J = 7.2 Hz, 1H, H2), 7.82 (d, J = 7.7 Hz, 1H, H5), 7.75 – 7.57 (m, 3H, H11, H12 and H13), 7.44 (t, J = 7.4 Hz, 1H, H3), 7.25 (t, J = 7.5 Hz, 1H, H4), 1.83 (s, 15H, Me-Cp*). ^{13}C NMR (75 MHz, CD2Cl2) δ 175.01 (C1), 163.11 (C7 or C8), 162.77 (C8 or C7), 135.19 (C5), 134.99 (C6), 133.61 (C3), 132.58 (C12), 131.83 (C9), 129.24 (C11 and C13), 127.83 (C10 and C14), 127.40 (C2), 123.21 (C4), 93.71 (x5, Ar-Cp*), 8.68 (x5, Me-Cp*). HRMS (ESI) m/z: $[M+H]^+$ Calcd for $C_{24}H_{25}ClIrN_4$ 597.1397. Found 597.1392; $[M-Cl]^+$ Calcd. for $C_{24}H_{24}IrN_4$ 561.1630 Found 561.1620.

Ir-C,N$_{Ph,Me}$. Reaction time: 1 day at RT. Column chromatography conditions: SiO_2, (DCM 100% -> DCM:MeOH 100:2). Obtained as brown powder. Yield: 98% ^{1}H NMR (300 MHz, CD_2Cl_2) δ 8.30 (dd, J = 7.7, 1.4 Hz, 1H, H2), 7.77 (d, J = 7.7 Hz, 1H, H5), 7.39 (td, J = 7.5, 1.5 Hz, 1H, H3), 7.28 – 7.12 (m, 1H, H4), 3.08 (s, 3H, H9), 1.75 (s, 15H, Me-Cp*). ^{13}C NMR (75 MHz, CD_2Cl_2) δ 175.04 (C1), 166.13 (C7 or C8), 162.44 (C8 or C7), 135.19 (C5), 134.89 (C6), 133.46 (C3), 127.11 (C2), 123.06 (C4), 93.54 (x5, Ar-Cp*), 20.68 (Me), 8.49 (x5, Me-Cp*). HRMS (ESI) m/z: $[M+H]^+$ Calcd for $C_{19}H_{23}ClIrN_4$ 535.1241. Found 535.1235. $[M-Cl]^+$ Calcd. for $C_{19}H_{22}IrN_4$ 499.1473. Found 499.1468.

Synthesis of BCN-FAM

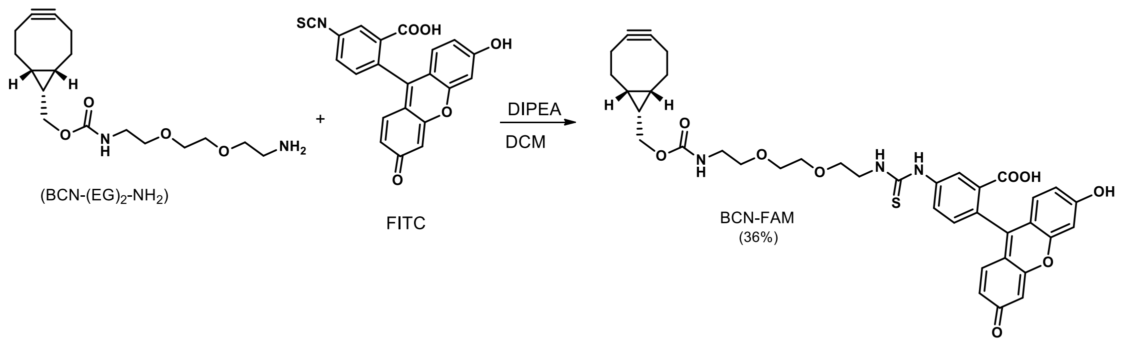

BCN-FAM was synthesized from a procedure adapted from the literature.[3] N,N-Diisopropylethylamine (DIPEA) and (1R,8S,9S)-Bicyclo[6.1.0]non-4-yn-9-ylmethyloxycarbonyl]-1,8-diamino-3,6-dioxaoctane (**BCN-(EG)$_2$-NH$_2$**, 0.077 mmol, 25 mg) were stirred in DCM (1 mL) for 1 h. After, a solution of fluorescein isothiocyanate (isomer I, 30 mg, 0.077 mmol) in 1 mL of DCM added and the reaction mixture was stirred at rt for 3 h. At the end of the reaction time, the solvent was evaporated under vacuum and the product was purified by preparative RP-HPLC on a 250 x 21 mm Nucleodur 18 HTec, 5 µm column (Macherey-Nagel) using 40% MeCN in 10 mM ammonium acetate at 10 ml/min with detection set at 490 nm. Fractions containing the pure compound were manually collected and freeze-dried to obtain the product as a yellow solid. ^{1}H NMR (400 MHz, MeOD) δ 8.12 (d, *J* = 1.6 Hz, 1H, H6), 7.82 (dd, *J* = 8.1 Hz, 1.6 Hz, 1H, H5), 7.19 (d, *J* = 8.2 Hz, 1H, H4), 6.87 (d, *J* = 8.9 Hz, 2H, H3), 6.70 (d, *J* = 2.3 Hz, 2H, H1), 6.60 (dd, *J* = 8.8, 2.3 Hz, 2H, H2), 4.12 (d, *J* = 8.1 Hz, 2H), 3.85 (s, 2H), 3.74 (t, *J* = 5.1 Hz, 2H), 3.69 (d, *J* = 3.5 Hz, 4H), 3.56 (t, *J* = 5.5 Hz, 2H), 3.29 (t, *J* = 5.5 Hz, 2H), 2.35 – 2.05 (m, 6H), 1.58 (d, *J* = 10.2 Hz, 2H), 1.46 – 1.25 (m, 2H), 0.92 (t, *J* = 9.3 Hz, 2H). HRMS (ESI) m/z: $[M+Na]^+$ Calcd for $C_{38}H_{39}N_3O_9SNa$ 736.2299. Found 736.2303.

References

[1] H. Xiong, Y. Gu, S. Zhang, F. Lu, Q. Ji, L. Liu, P. Ma, G. Yang, W. Hou, H. Xu, *Chem. Commun.* **2020**, *56*, 4692–4695.

[2] S. Eising, B.-T. Xin, F. Kleinpenning, J. J. A. Heming, B. I. Florea, H. S. Overkleeft, K. M. Bonger, *Chembiochem Eur. J. Chem. Biol.* **2018**, *19*, 1648–1652.

[3] P. M. S. D. Cal, R. F. M. Frade, C. Cordeiro, P. M. P. Gois, *Chem. – Eur. J.* **2015**, *21*, 8182–8187.

Supplementary Figures

NMR spectra

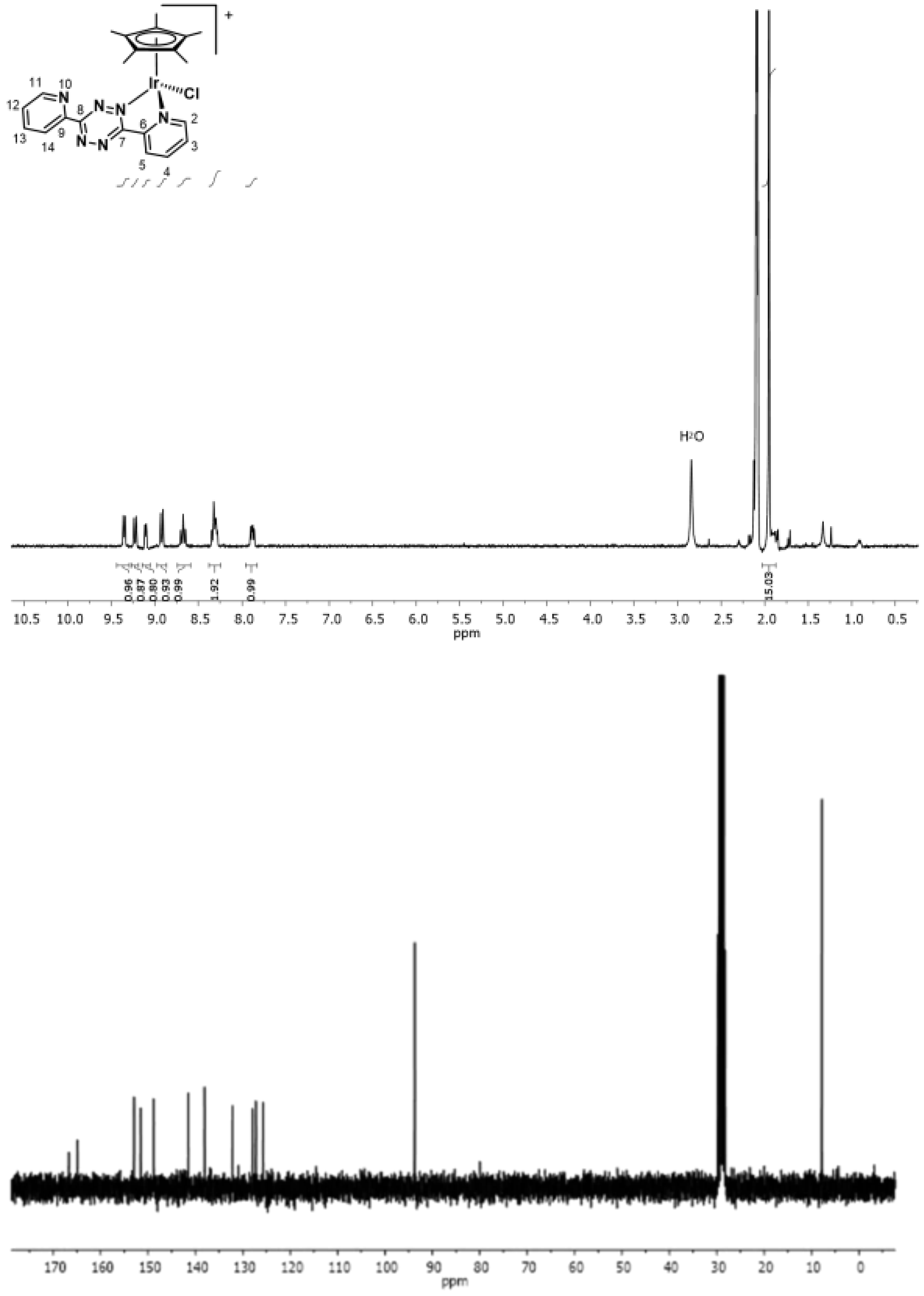

Figure S 1. ^{1}H NMR (400 MHz, Acetone-d_6, 298 K) and ^{13}C NMR (100 MHz, Acetone-d_6, 298 K) spectra of Ir-N,$N_{Py,Py}$.

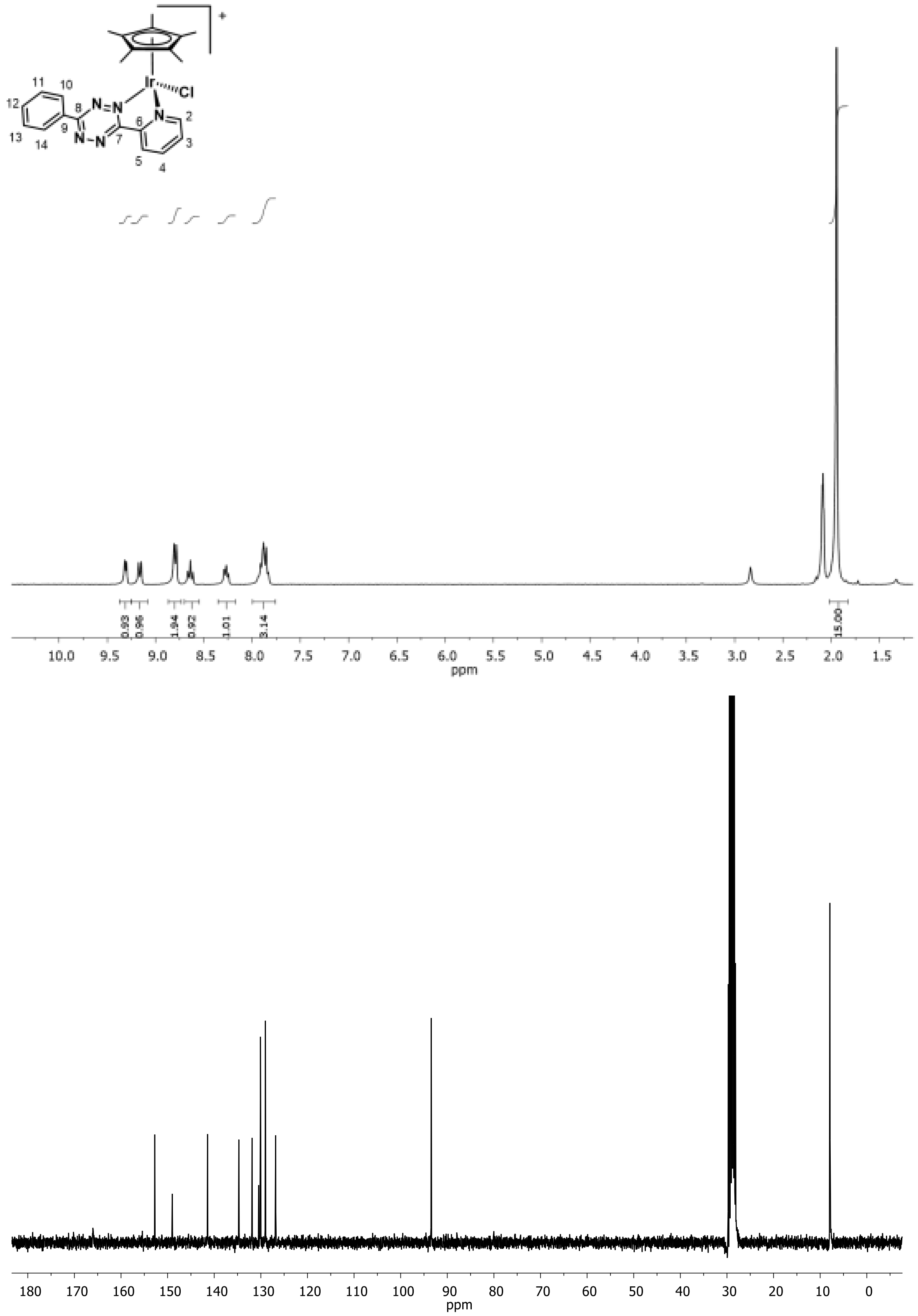

Figure S 2. ^{1}H NMR (400 MHz, Acetone-d_6, 298 K) and ^{13}C NMR (100 MHz, Acetone-d_6, 298 K) spectra of Ir-N,$N_{Py,Ph}$.

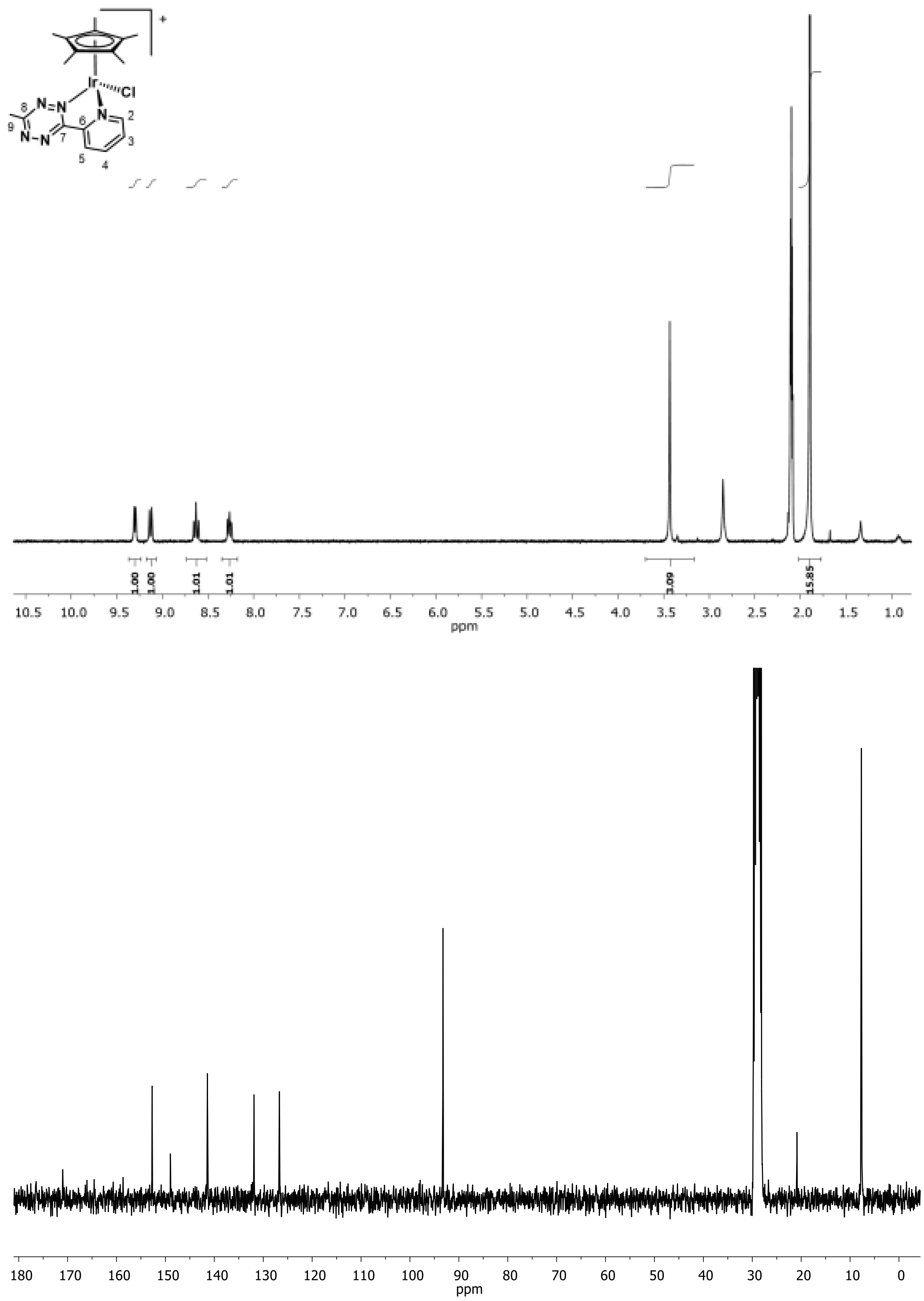

Figure S 1. ^{1}H NMR (400 MHz, Acetone-d_6, 298 K) and ^{13}C NMR (100 MHz, Acetone-d_6, 298 K) spectra of Ir-N,$N_{Py,Me}$.

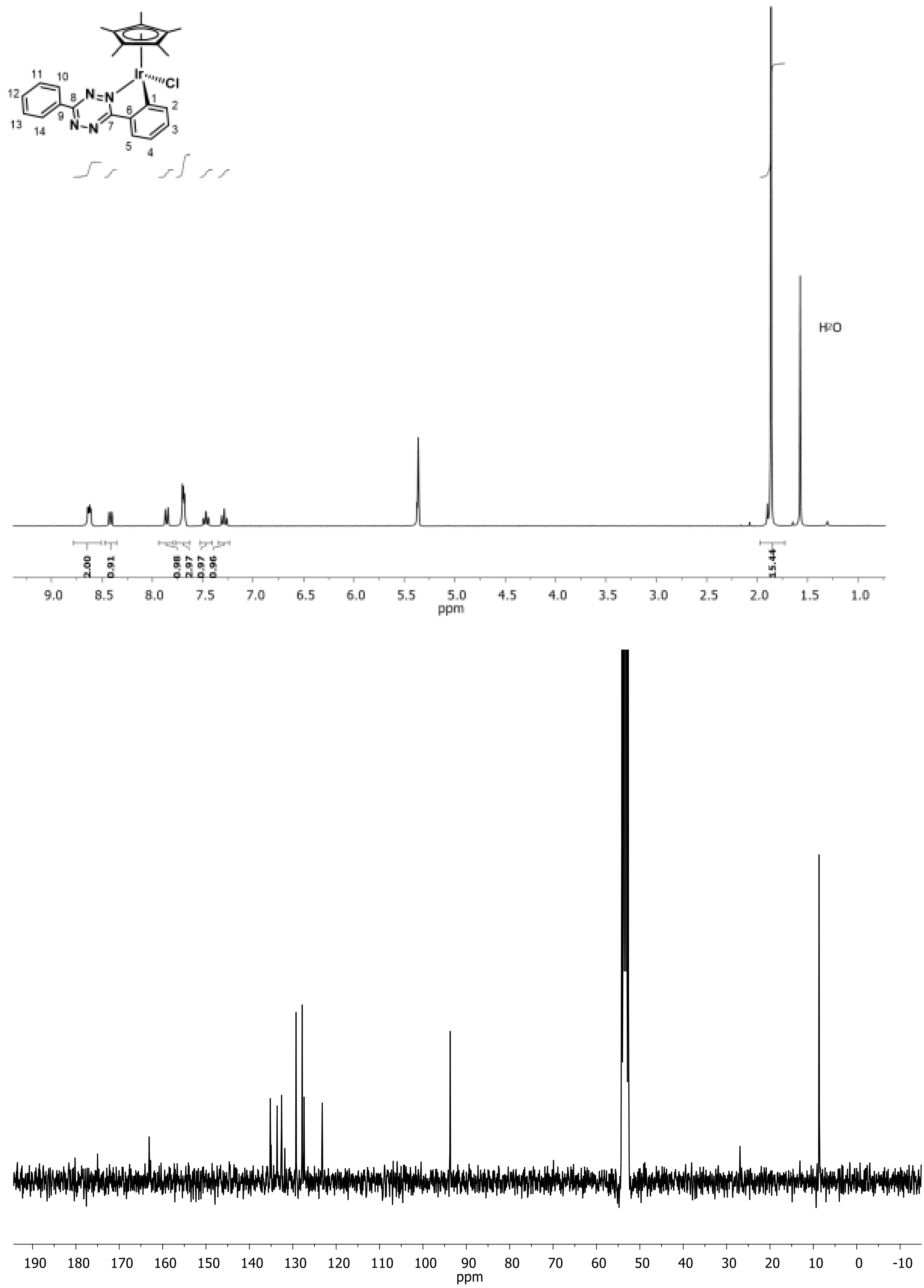

Figure S 2. ^{1}H NMR (400 MHz, DCM-d_2, 298 K) and ^{13}C NMR (100 MHz, DCM-d_2, 298 K) spectra of Ir-C,$N_{Ph,Ph}$.

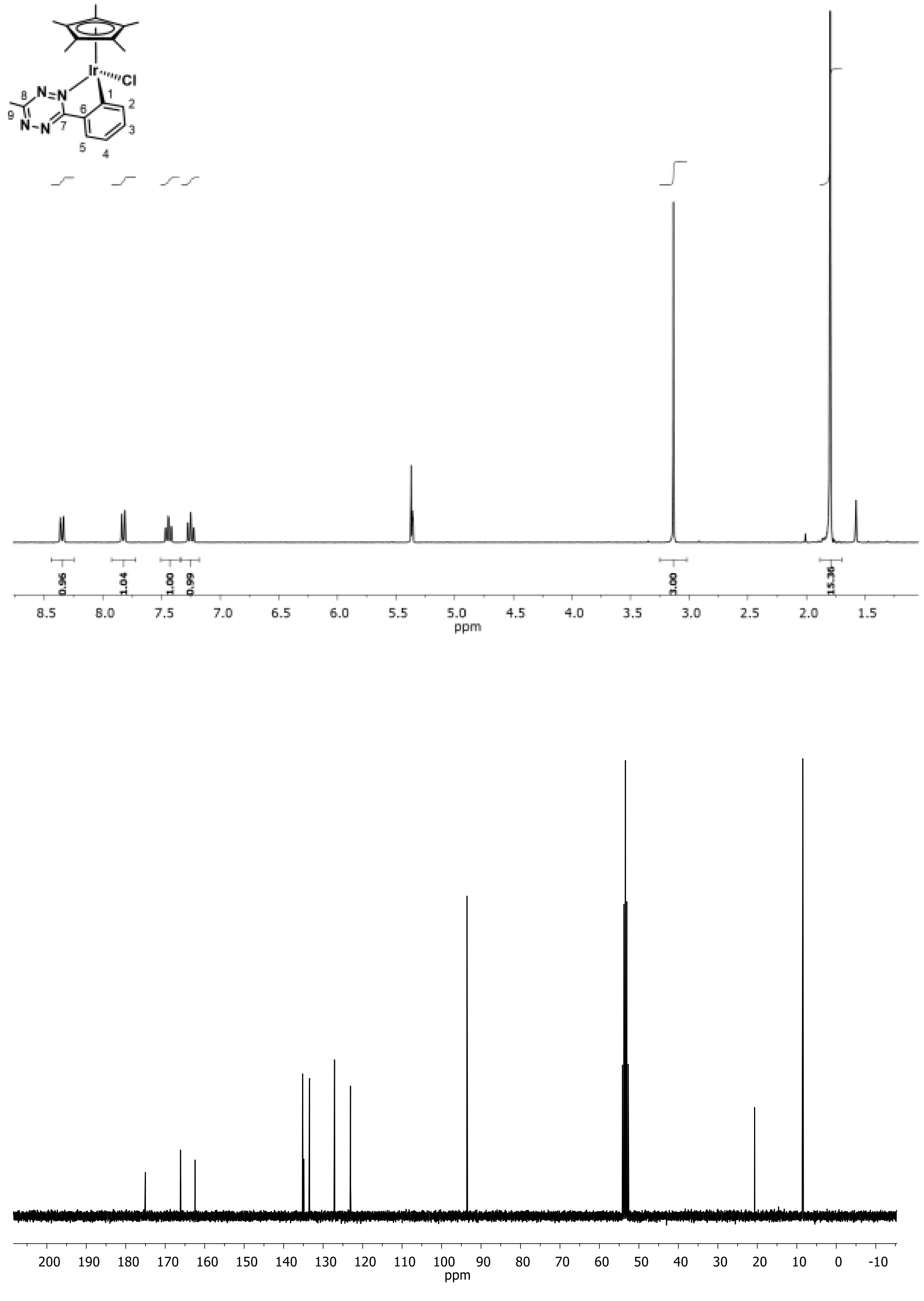

Figure S 3. ^{1}H NMR (400 MHz, DCM-d_2, 298 K) and ^{13}C NMR (100 MHz, DCM-d_2, 298 K) spectra of Ir-C,$N_{Ph,Me}$.

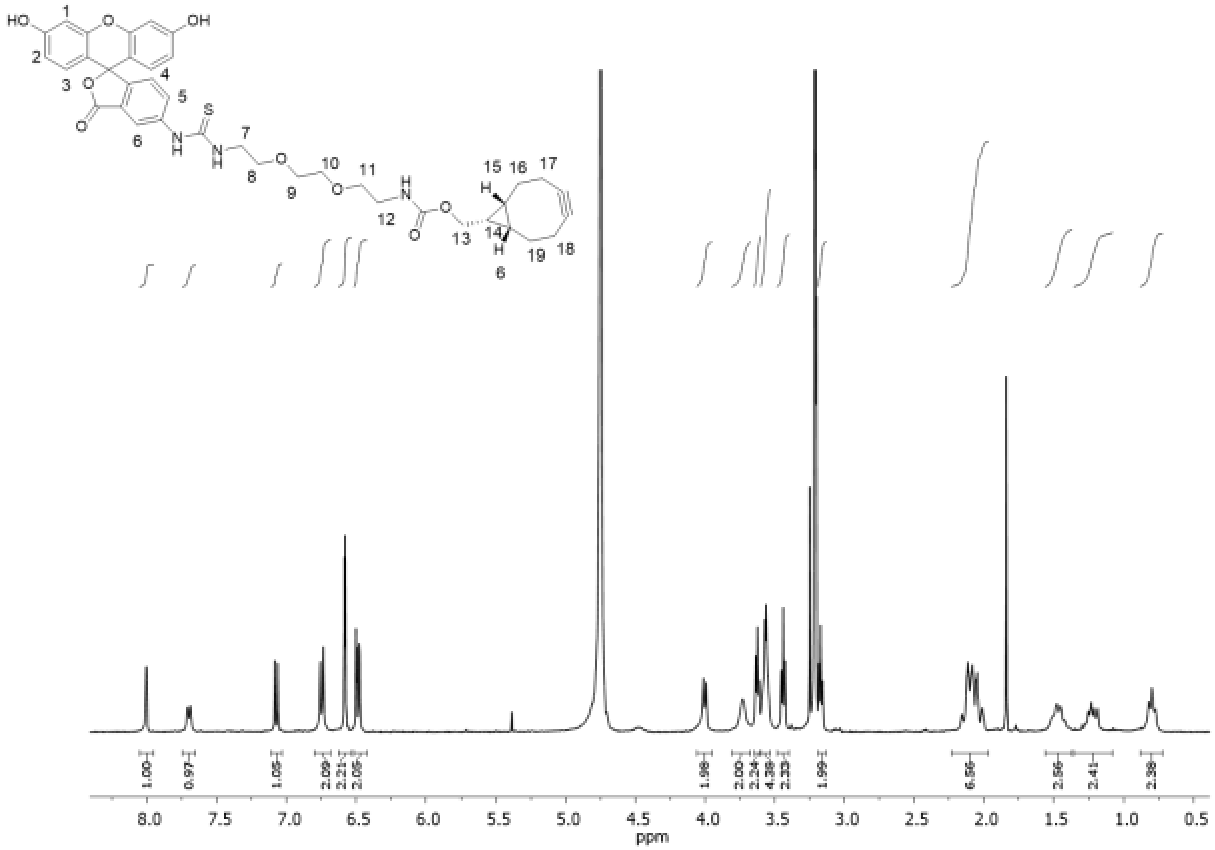

Figure S 4. ^{1}H NMR (400 MHz, MeOD-d_4, 298 K) spectrum of BCN-FAM

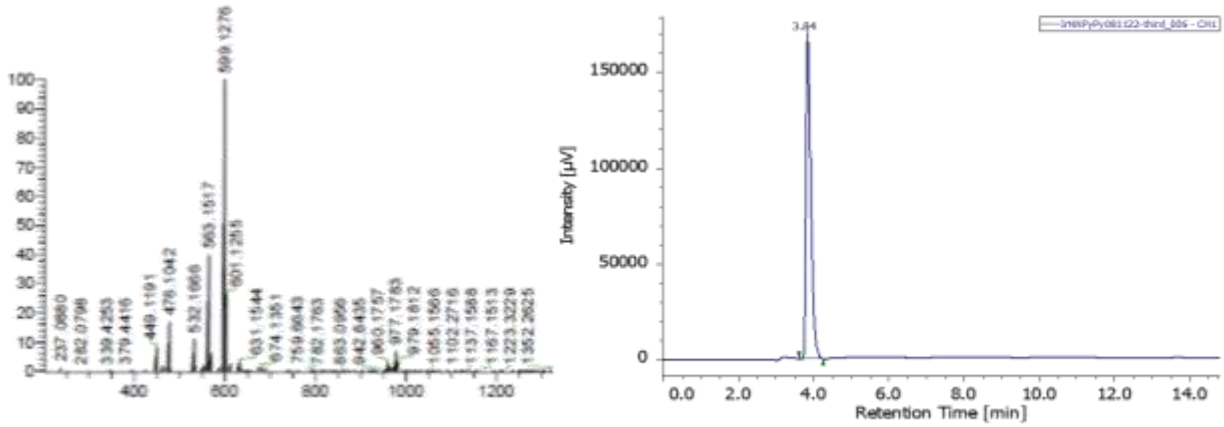

Figure S 7. HRMS-ESI spectrum and HPLC chromatogram of Ir-N,$N_{Py,Py}$ (30 µM in MeCN). HPLC conditions 1-1

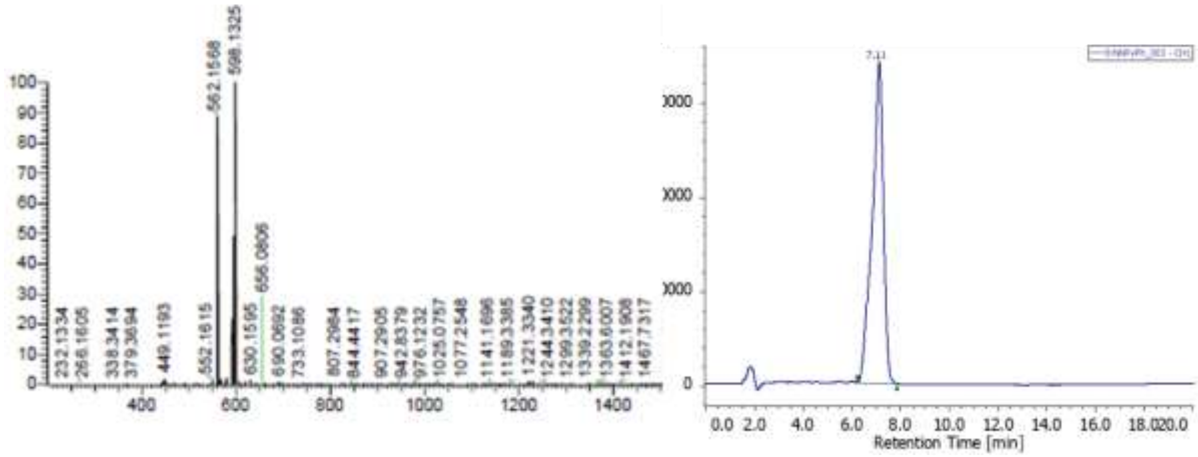

Figure S 5. HRMS-ESI spectrum and HPLC chromatogram of Ir-N,$N_{Py,Ph}$ (30 µM in MeCN). HPLC conditions 1-2

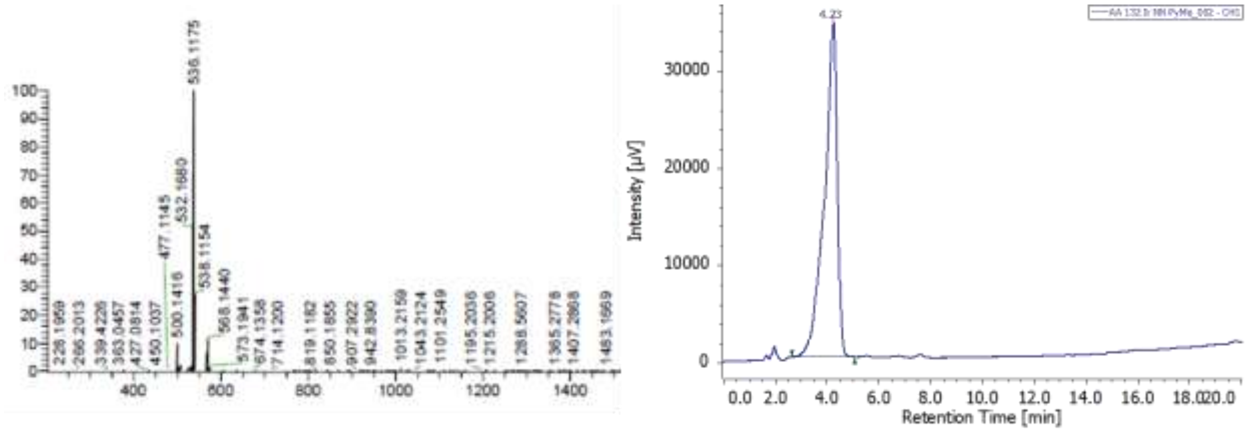

Figure S 9. HRMS-ESI spectrum and HPLC chromatogram of Ir-N,$N_{Py,Me}$ (30 µM in MeCN). HPLC conditions 1-5

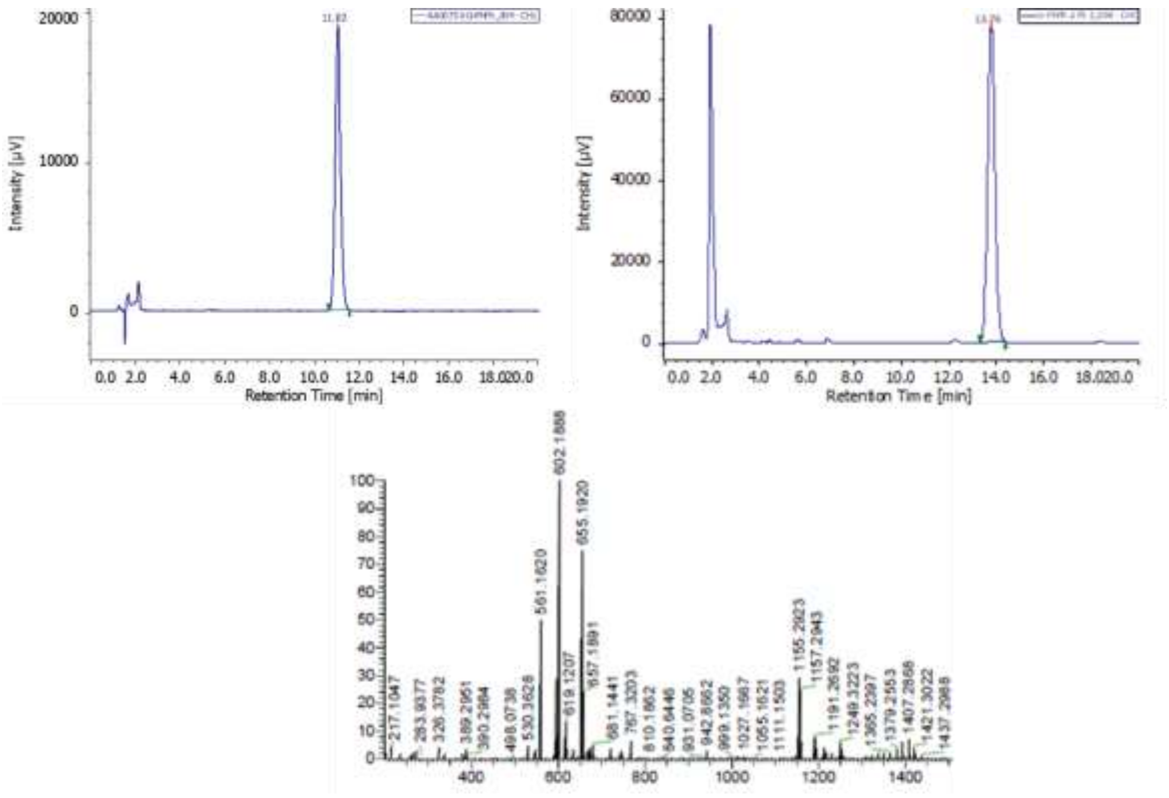

Figure S 10. HRMS-ESI spectrum and HPLC chromatograms of Ir-C,$N_{Ph,Ph}$ (30 µM in MeCN (left) or DMSO (right)). HPLC conditions 1-4

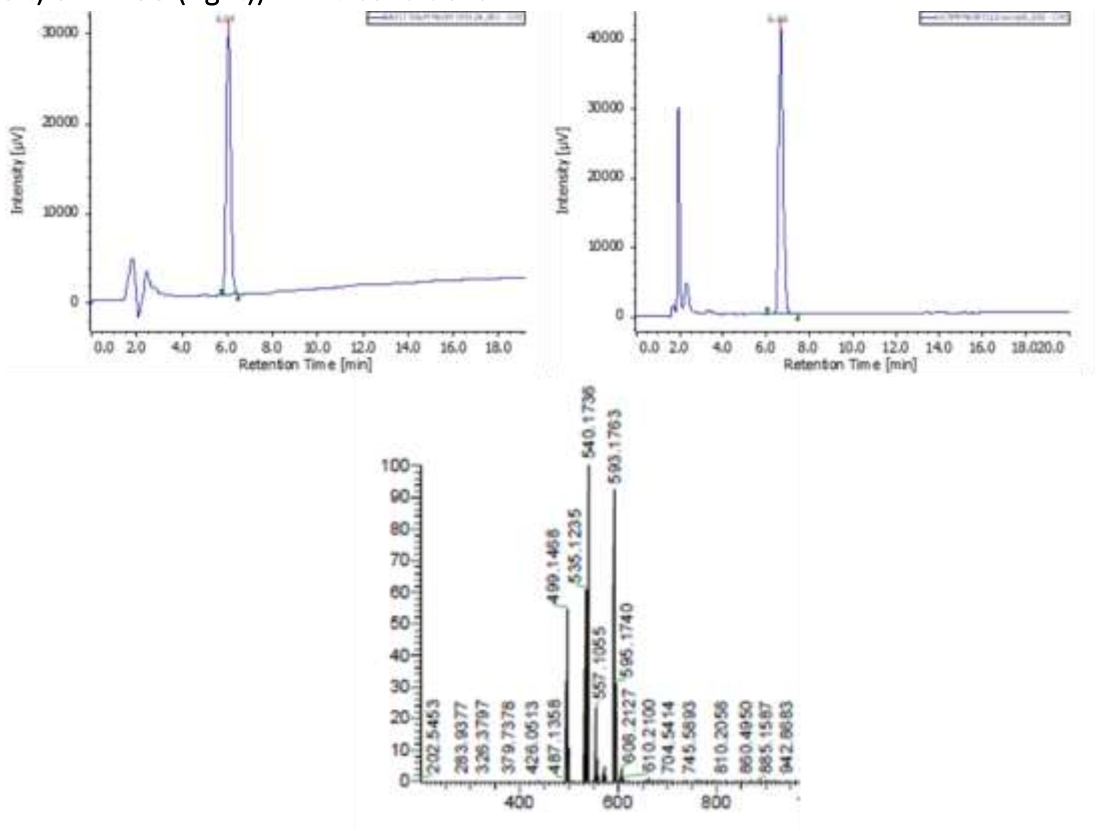

Figure S 11. HRMS-ESI spectrum and HPLC chromatograms of Ir-C,$N_{Ph,Me}$ (30 µM in MeCN (left) or DMSO (right)). HPLC conditions 1-4

In-solution NMR studies

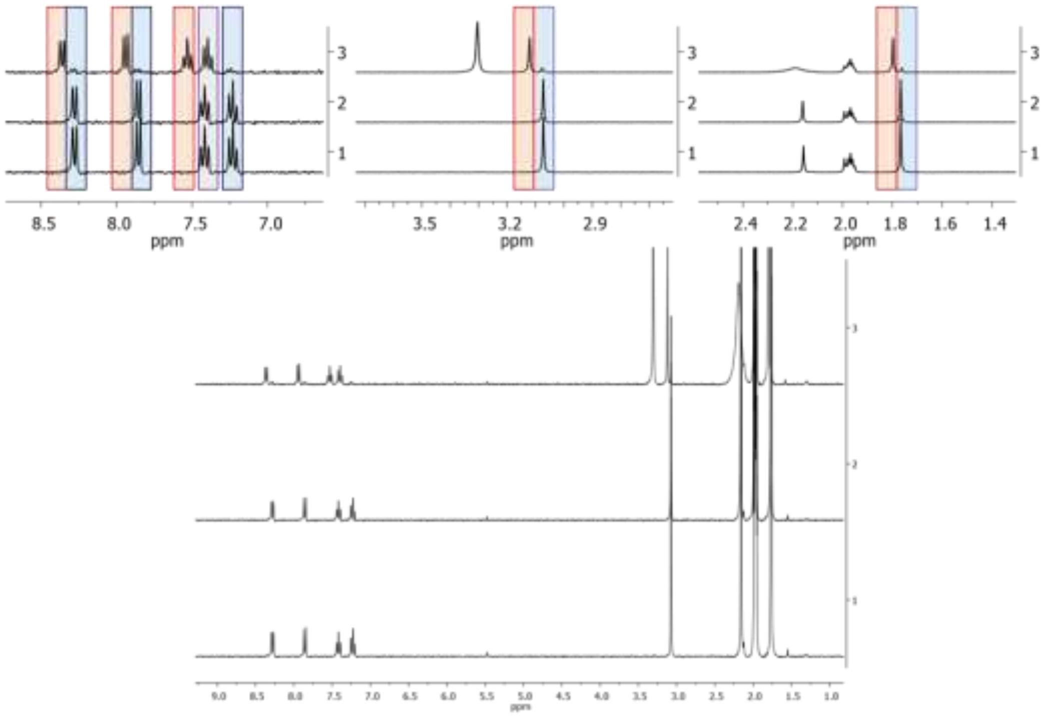

Figure S 6. ^{1}H NMR spectra of Ir-C,N$_{Ph,Me}$ in MeCN-d$_3$

Spectra recorded immediately after dissolution (trace 1), after 24 h (trace 2), after addition of $AgBF_4$ (trace 3); (blue) **Ir-C,N$_{Ph,Me}$** (red) [**Ir-C,N$_{Ph,Me}$-MeCN**]$^+$; spectra recorded at 300 MHz, 298 K.

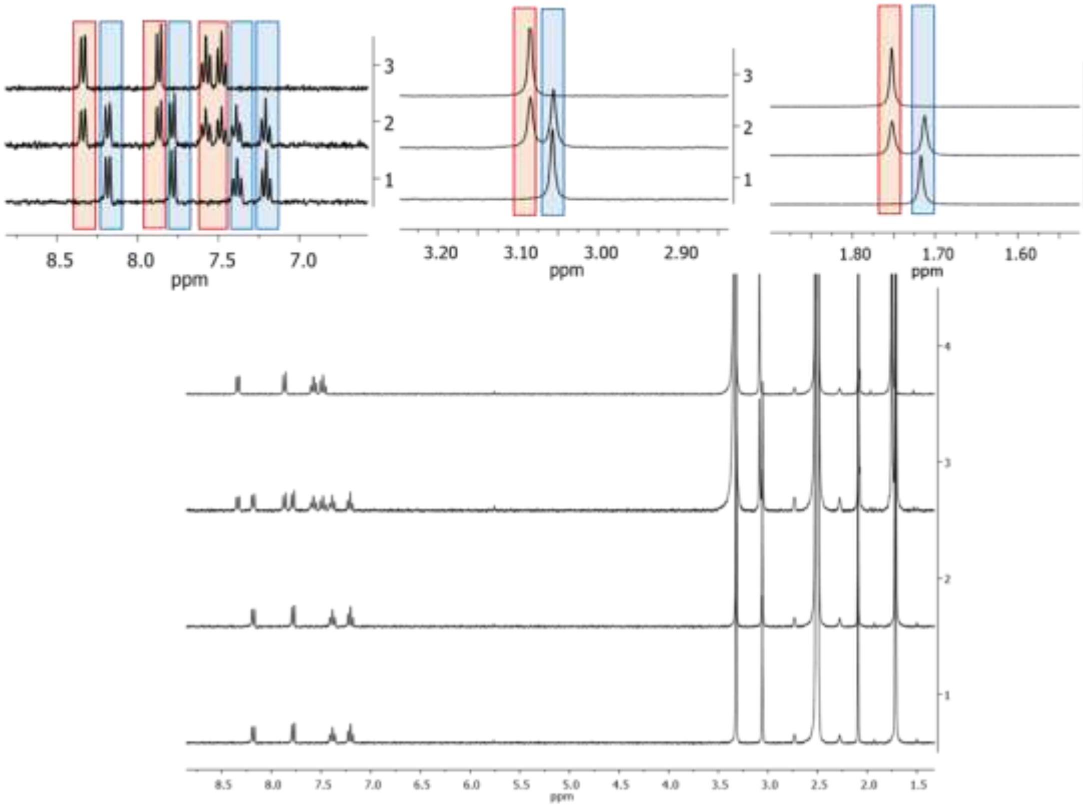

Figure S 13. 1H NMR spectra of Ir-C,NPh,Me in DMSO-d$_6$

Spectra recorded immediately after dissolution (trace 1), after 24 h (trace 2), after addition of $AgBF_4$ (trace 3); (in blue) **Ir-C,N$_{Ph,Me}$,** (red) [**Ir-C,N$_{Ph,Me}$-MeCN**]$^+$; spectra recorded at 300 MHz, 298 K.

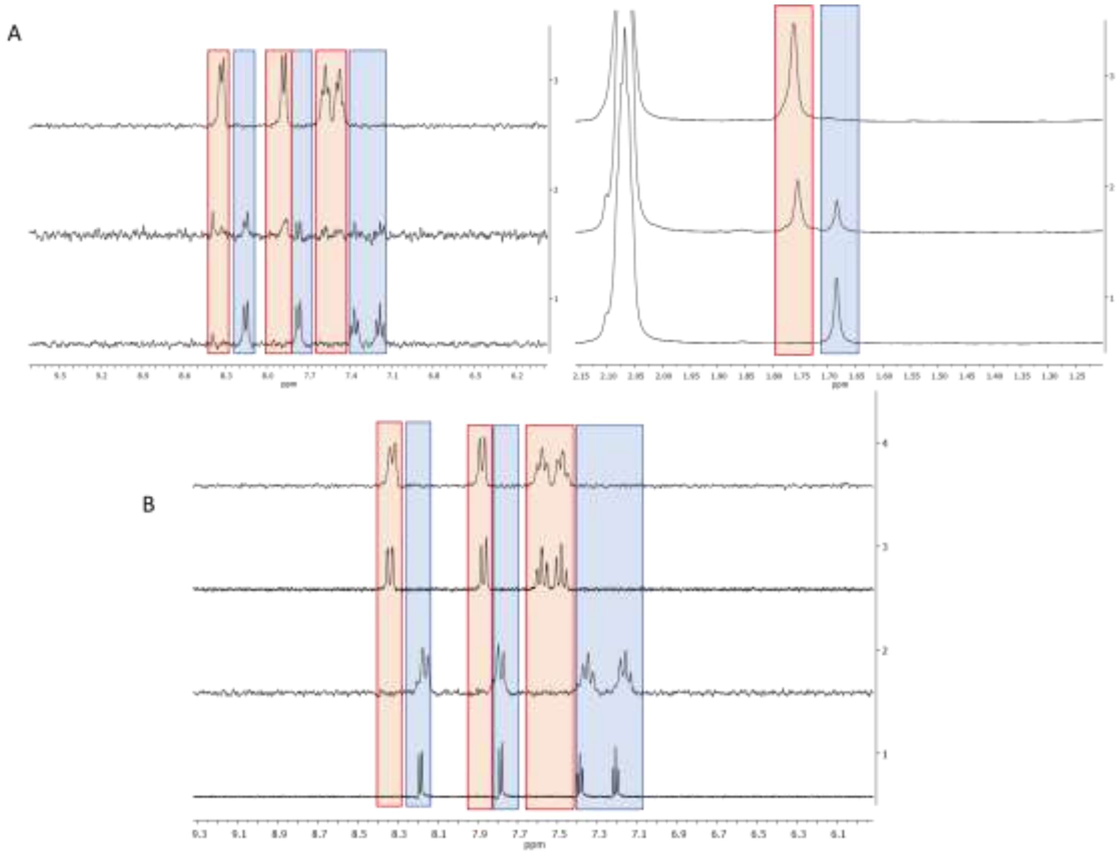

Figure S 7. ^{1}H NMR spectra of of Ir-C,$N_{Ph,Me}$ in acetone-d_6/D_2O/DMSO-d_6 4:3:3.

Spectra recorded immediately after dissolution (trace 1), after 24 (trace 2) and 48 h (trace 3); B) ^{1}H NMR spectra of **Ir-C,$N_{Ph,Me}$** in DMSO-d_6 (trace 1), in D_2O/Acetone-d_6 1:1 (trace 2), in DMSO-d_6 after addition of $AgBF_4$ to generate [**Ir-C,$N_{Ph,Me}$-DMSO**]$^+$ (trace 3) and in acetone-d_6/D_2O/DMSO-d_6 in 4:3:3 after 48 h (trace 4). **Ir-C,$N_{Ph,Me}$** (in blue), [**Ir-C,$N_{Ph,Me}$-DMSO**]$^+$ (in red) spectra recorded at 300 MHz, 298 K. Comparison of traces 1 and 2 shows the presence of the chlorido complex in DMSO-d_6 and D_2O/Acetone-d_6 1:1 (immediately after dissolution). Comparison of traces 3 and 4 demonstrates the *in situ* formation of the DMSO-adduct in DMSO-d_6 by addition of $AgBF_4$ or formed in acetone-d_6/D_2O/DMSO-d_6 in 4:3:3 over 48 h at RT

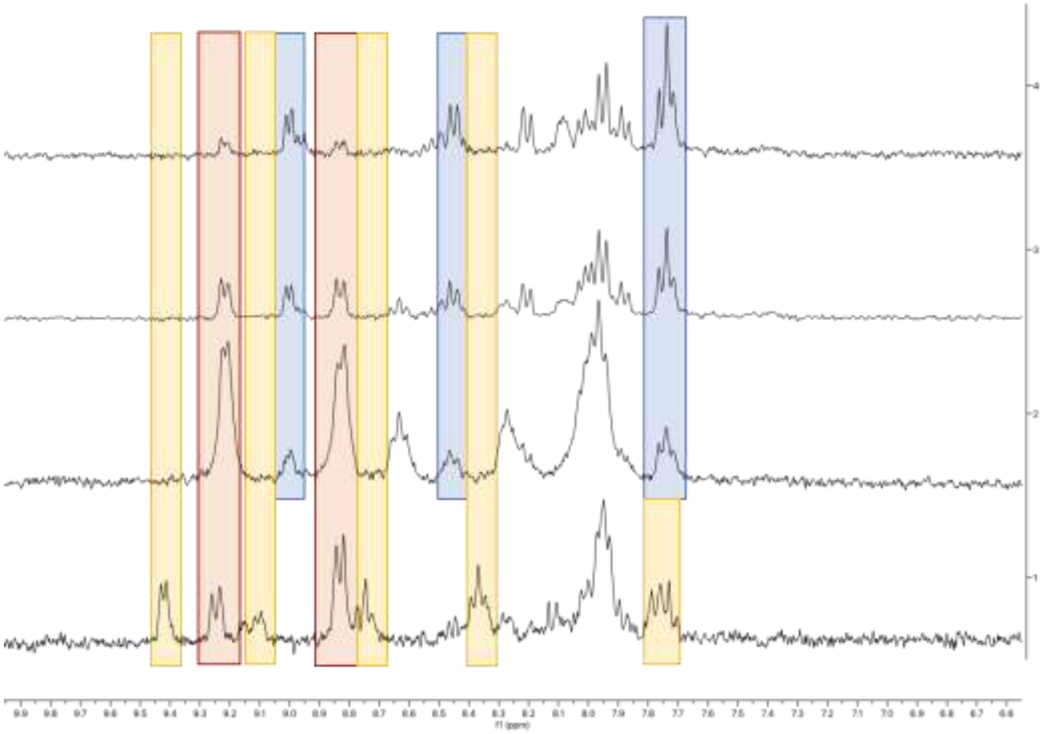

Figure S 8. ^{1}H NMR spectra of Ir-N,$N_{Py,Ph}$ in acetone-d_6-D_2O 1:1

After 24 h (trace 1) and in DMSO-d_6- D_2O 1:1 after 30 min (trace 2), 24 h (trace 3), 4 days (trace 4). **Ir-N,$N_{Py,Ph}$** (red), [**Ir-N,$N_{Py,Ph}$-DMSO**]$^{2+}$ (blue) and [**Ir-N,$N_{Py,Ph}$-D_2O**]$^{2+}$ (yellow); spectra recorded at 300 MHz, 298 K. No clear traces of the aqua-species were observed after 24 h in DMSO-d_6- D_2O 1:1.

iEDDA Reactivity with BCN-OH

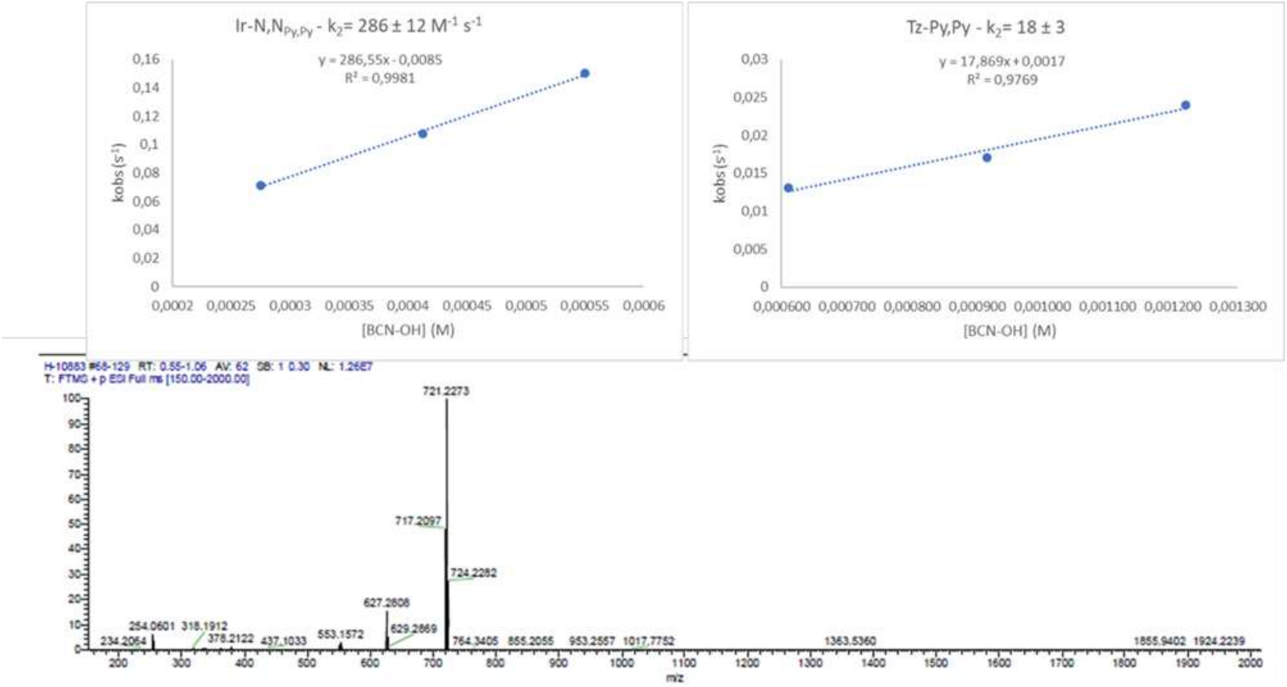

Figure S16. Kinetics of reaction of Ir-N,N$_{Py,Py}$ and BCN-OH in MeCN at 24 °C.

Plots of the k_{obs} values vs. [BCN-OH] for the Ir-complex and the non-coordinated tetrazine. On the bottom the HRMS-ESI spectrum of the product

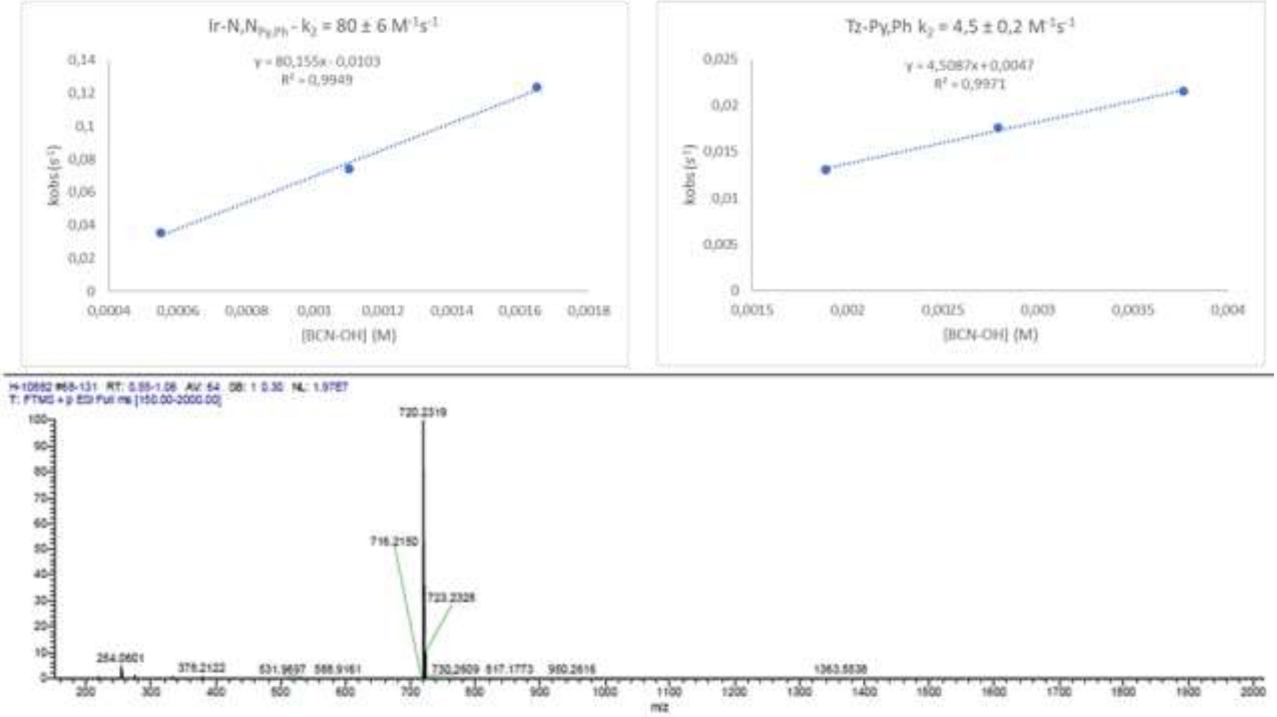

Figure S17. Kinetics of reaction of Ir-N,N$_{Py,Ph}$ and BCN-OH in MeCN at 24 °C.

Plots of the k_{obs} values vs. [BCN-OH] for the Ir-complex and the non-coordinated tetrazine. On the bottom the HRMS-ESI spectrum of the product

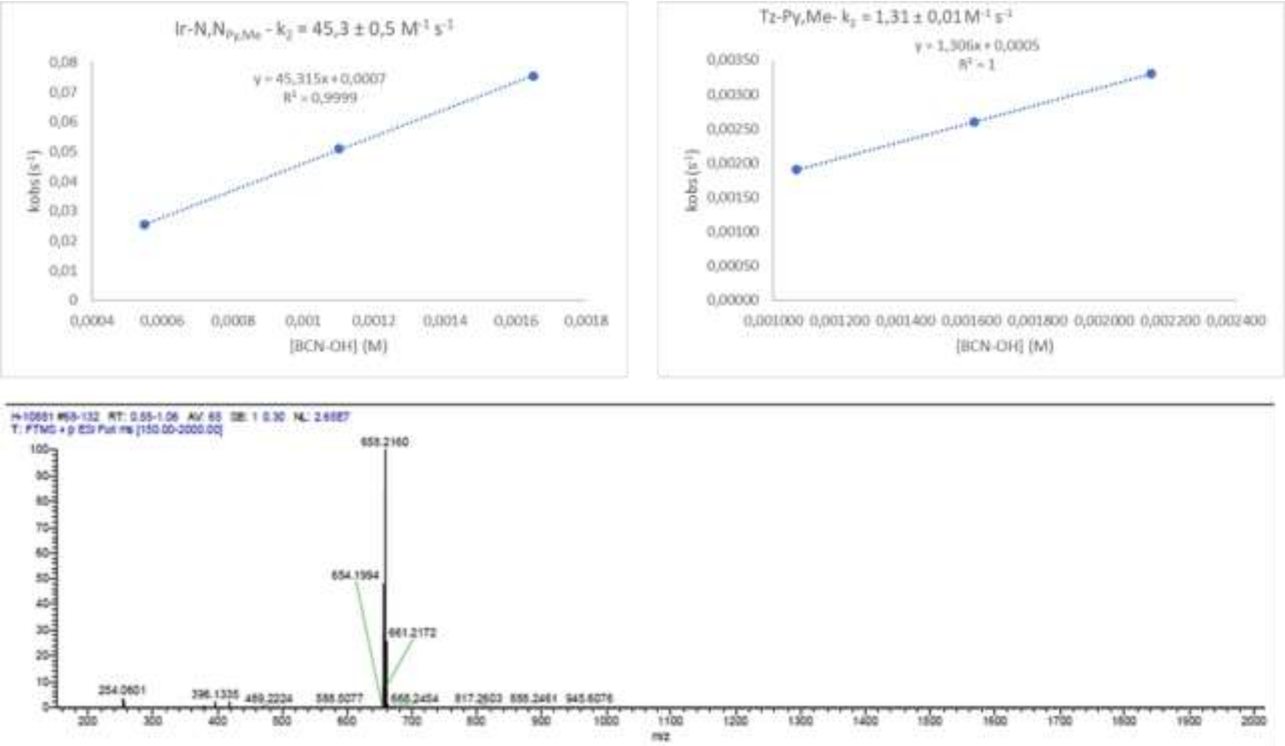

Figure S18. Kinetics of reaction of Ir-N,N$_{Py,Me}$ and BCN-OH in MeCN at 24 °C.

Plots of the k_{obs} values vs. [BCN-OH] for the Ir-complex and the non-coordinated tetrazine. On the bottom the HRMS-ESI spectrum of the product

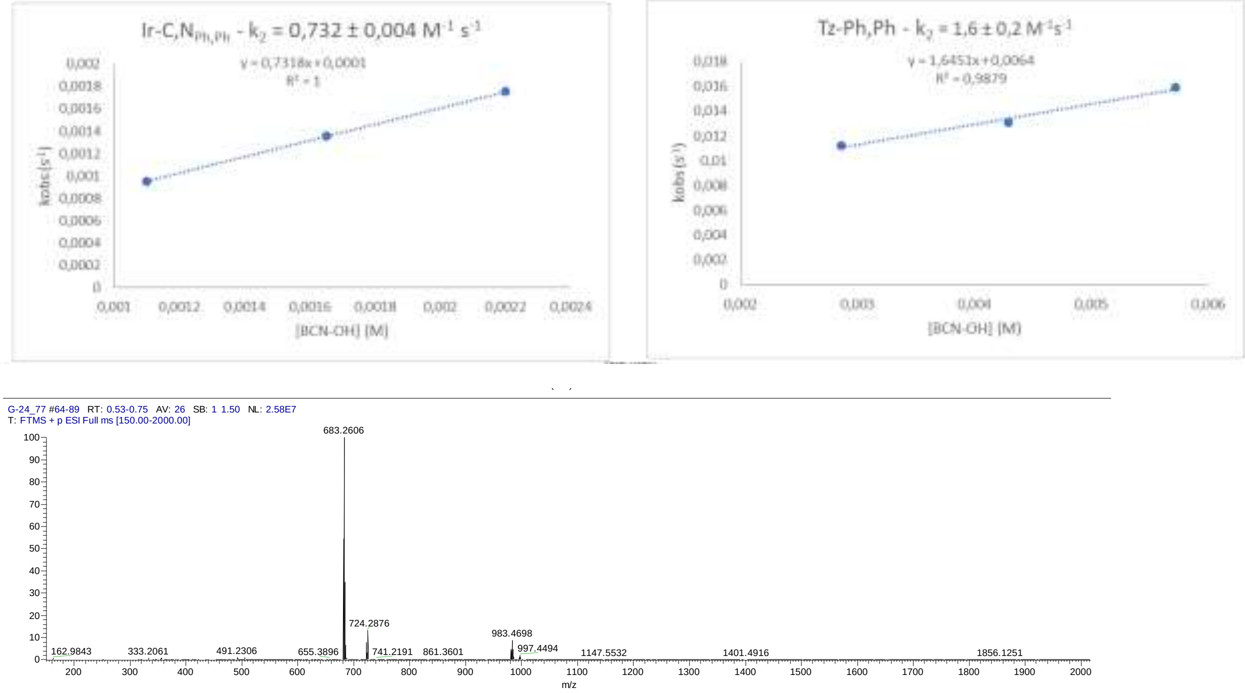

Figure S 19. Kinetics of reaction of Ir-C,N$_{Ph,Ph}$ and BCN-OH in MeCN at 24 °C.

Plots of the k_{obs} values vs. [BCN-OH] for the Ir-complex and the non-coordinated tetrazine measured. On the bottom the HRMS-ESI spectrum of the product

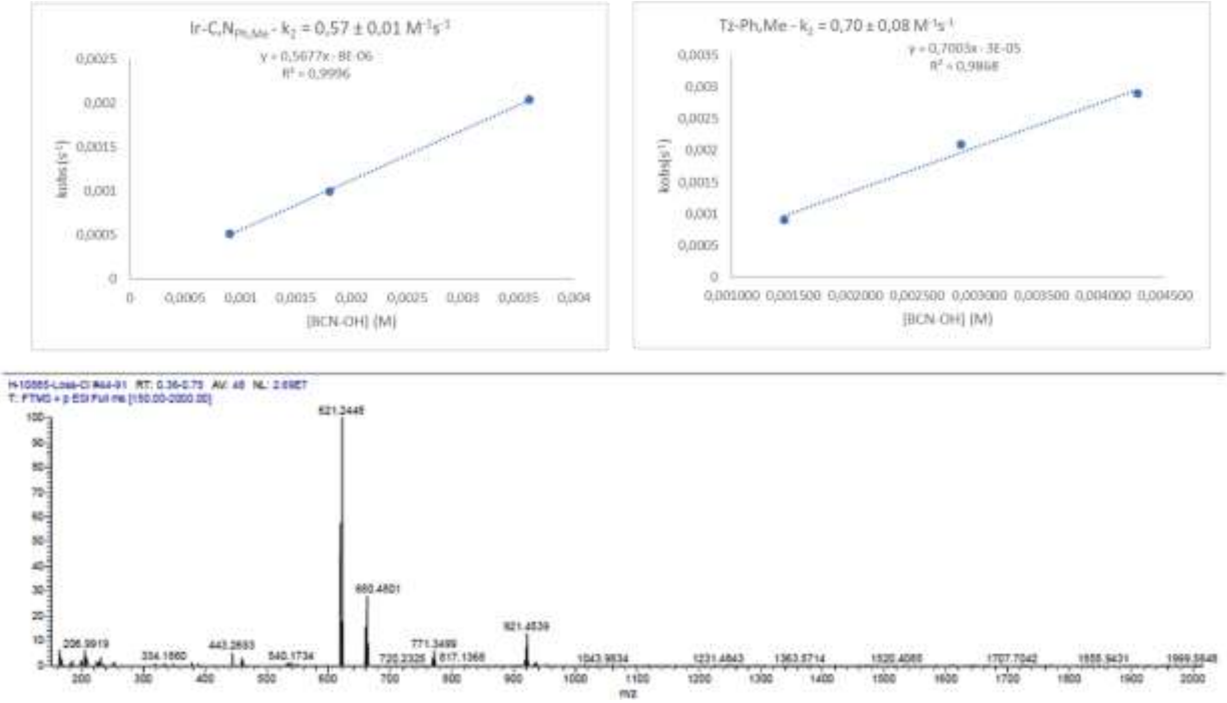

Figure S 20. Kinetics of reaction of Ir-C,N$_{Ph,Me}$ and BCN-OH in MeCN at 24 °C.

Plots of the k_{obs} values vs. [BCN-OH] for the Ir-complex and the non-coordinated tetrazine. On the bottom the HRMS-ESI spectrum of the product

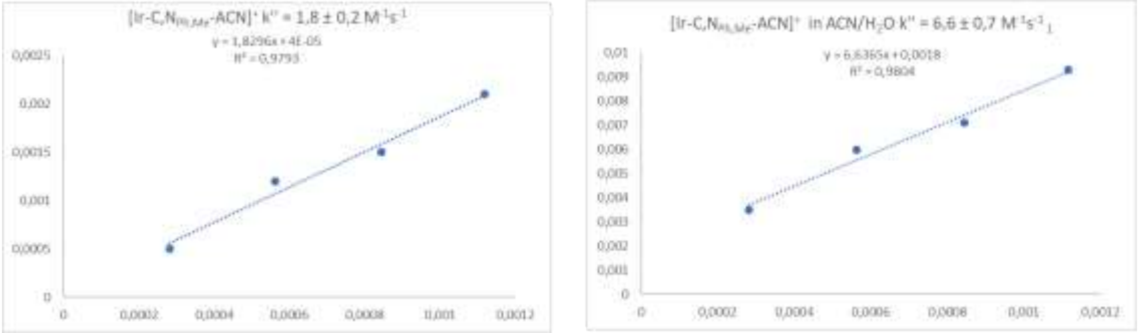

Figure S 21. Kinetics of reaction of Ir-C,N$_{Ph,Me}$ and BCN-OH.

Plots of the k_{obs} values vs. [BCN-OH] for [Ir-C,N$_{Ph,Me}$ -MeCN]$^+$ in MeCN (left) and in MeCN/H_2O 1:1 at 24 °C (right).

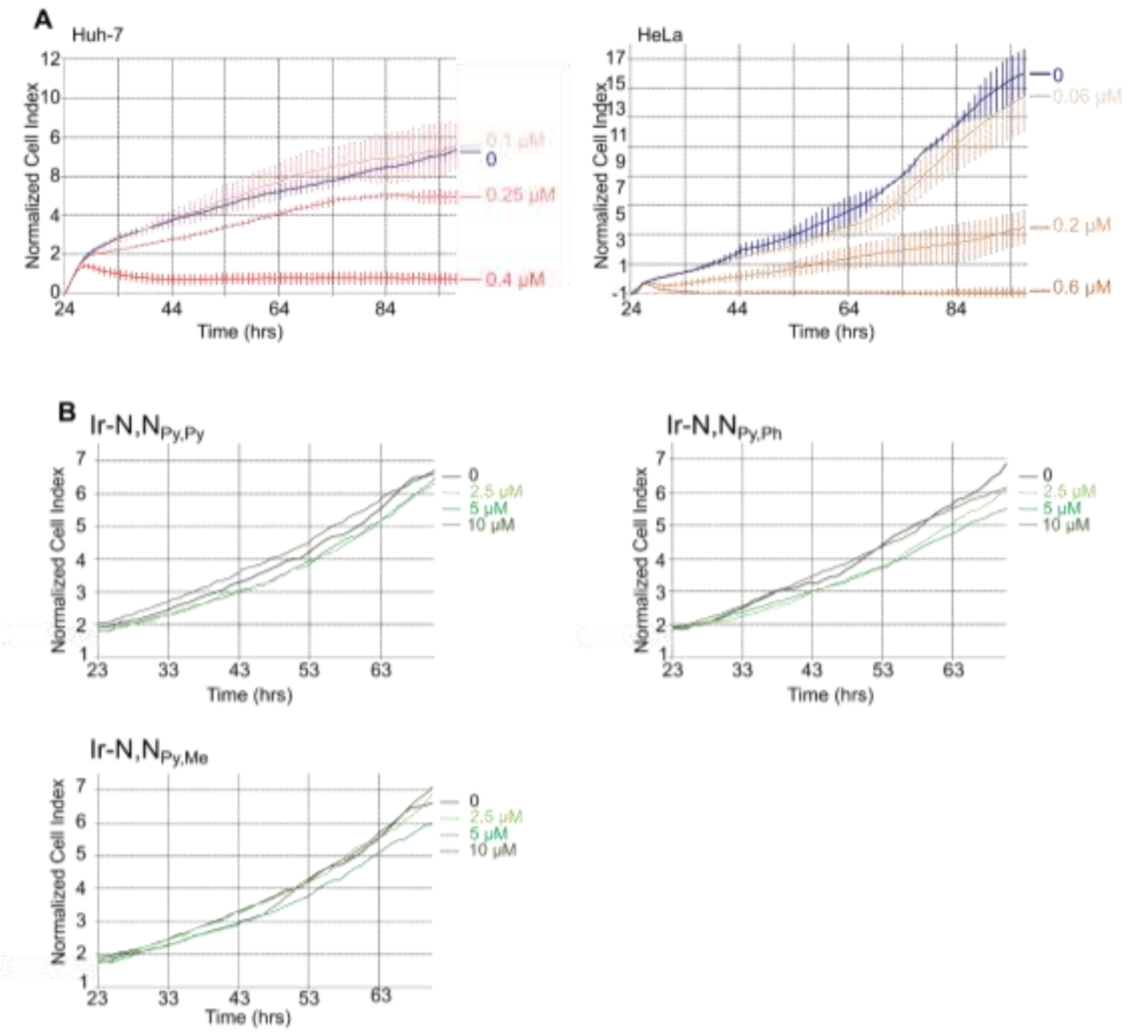

Figure S 22. Real time analysis of cells exposed to the iridium complexes.

(A) Huh-7 and HeLa cells were exposed to the indicated concentrations of **Ir-C,N,$_{Ph,Me}$** showing a dose-dependent inhibition of cell adhesion and proliferation. (B) Huh-7 cells were exposed to the indicated **Ir-N,N$_{Py,R}$** complex, showing the absence of biological effects up to 10 µM. Iridium complexes were added at time point 24 hrs (A) and 23 hrs (B).

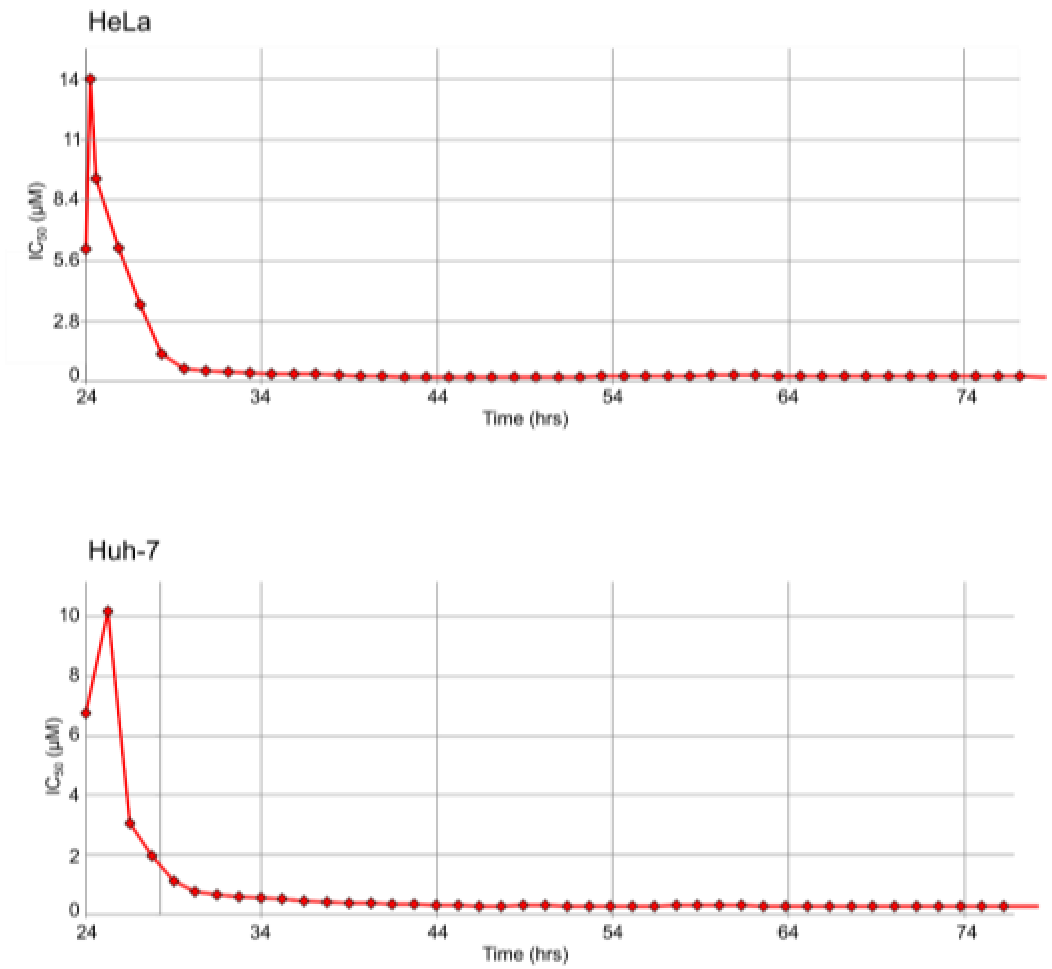

Figure S 23. Time dependent IC_{50} of Ir-C,$N_{,Ph,Me}$ in HeLa and Huh-7 cell lines

A stable value is reached within 24 h after addition of the complex to cells. Graphs are derived from the plots shown in Fig. S22.

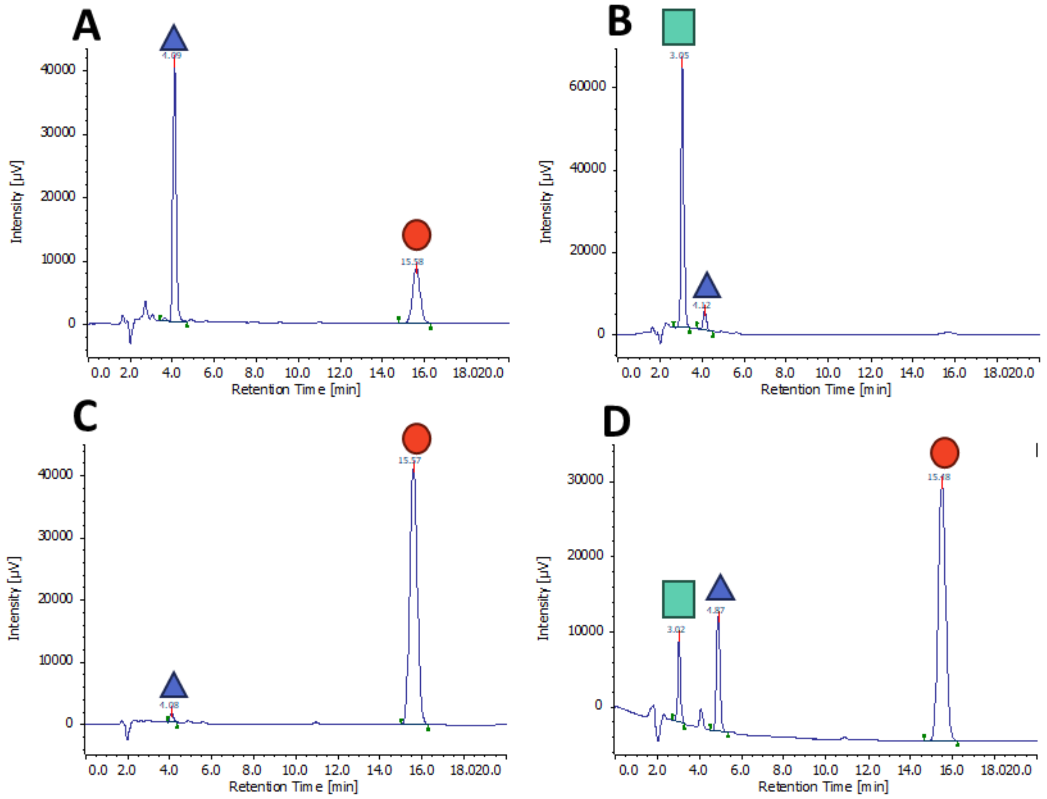

Figure S 24. HPLC analysis of Ir-C,$N_{Ph,Me}$ (50 µM) in H_2O:MeOH 95/5 (A,B) or PBS:MeOH 95/5 (C-D).

A) after 3 h at RT; (B) 40 min after addition of 1 mM DMSO, 37°C; (C) immediately after dilution; (D) immediately after addition of 1 mM DMSO. The peaks at 3.0 min (■), 4.1 min (▲) and 15.6 min (●) are respectively assigned to the DMSO, aquo and chlorido complexes. The proportions in each species are summarized in Table S1. As observed in trace B, the aqua complex is still observed. As mentioned in the main text, the lack of observation of the aqua-complex by NMR in DMSO-D_2O is probably due to the different sensitivity of the two techniques. HPLC conditions 1-3.

Table S 1. Speciation of Ir-C,$N_{Ph,Me}$ in different media according to HPLC chromatograms shown in Figure S24 and Figure 2

Solvent mixture	Cl complex (t_R = 15.5 min)	H_2O complex (t_R = 4.1 min)	DMSO complex (t_R = 3.0 min)
H_2O:MeOH 95/5, 0 h	89%	11%	-
H_2O:MeOH 95/5, 3 h	32%	68%	-
H_2O:MeOH 95/5 + 1 mM DMSO, 40 min, 37°C	0%	7%	93%
PBS:MeOH 95/5	98%	2%	-
PBS/MeOH 95/5 + 1 mM DMSO, 0 h	75%	9%	16%
PBS/MeOH 95/5 + 13 mM DMSO, 1 h, 37°C	-	-	100%
DMEM/DMSO 99.9/0.1, 0 min[a]	75%	0%	25%
DMEM/DMSO 99.9/0.1, 30 min	0%	0%	100%[b]

[a] 0.1% DMSO = 13 mM. The overall equilibrium among the three species is here represented in the different conditions. In PBS, at low percentage of DMSO, the aqua complex is observed as the predominant species.

Reaction with amino acids

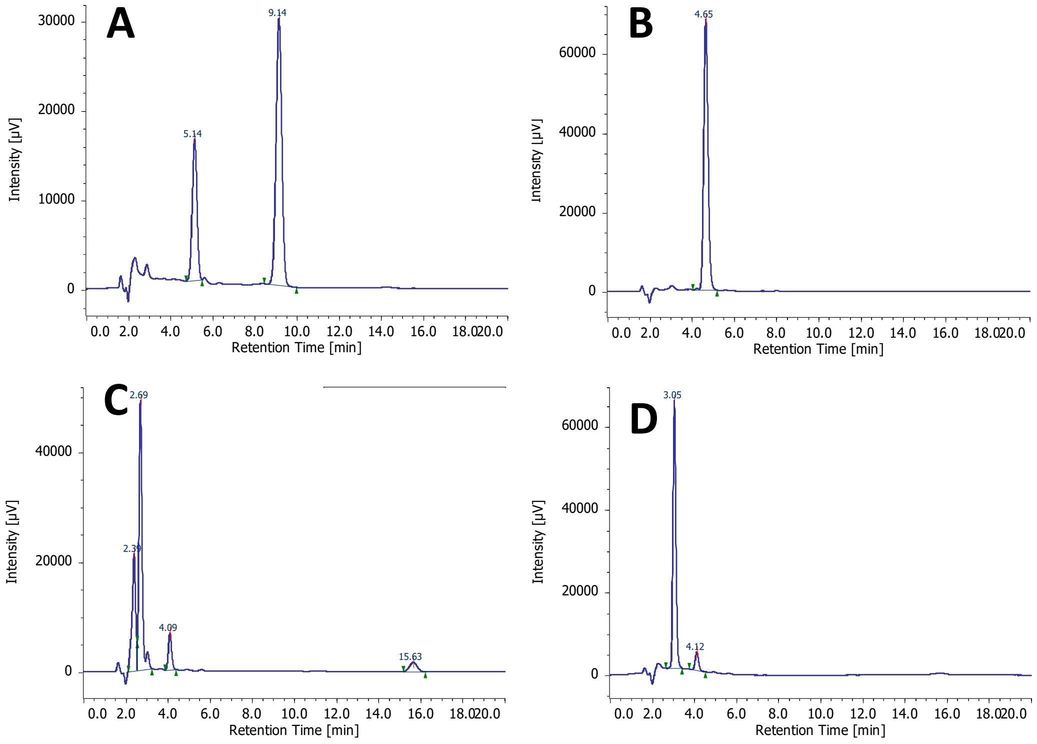

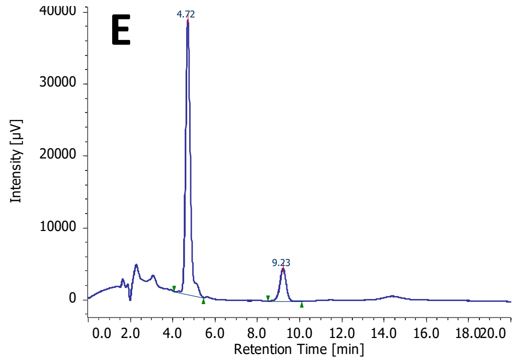

t_R (min)	assignment
15.6	Ir-C,$N_{Ph,Me}$
5.1 & 9.1	Cys adduct
4.1	Aqua complex
4.65	Met adduct
3.0	DMSO adduct
2.4 & 2.7	His adduct

Figure S 259. HPLC analysis of reaction mixtures of Ir-C,$N_{Ph,Me}$ with amino acid derivatives or DMSO.

Ir-C,$N_{Ph,Me}$ (50 µM) was incubated in H_2O/MeOH 93:7 with the following amino acids at 37 °C for 2 h. (A) N-acetyl-L-cysteine methyl ester (Cys, 325 µM), (B) N-Boc-L-methionine (Met, 325 µM), (C) N-acetyl-L-histidine (His, 325 µM), (D) DMSO (325 µM), (E) mixture of Cys, Met, His and DMSO (325 µM each). Chromatogram (E) presents 2 peaks respectively assigned to the Met and Cys adducts in the ratio 86 to 14, suggesting a preference for methionine over cysteine binding. HPLC conditions 1-3

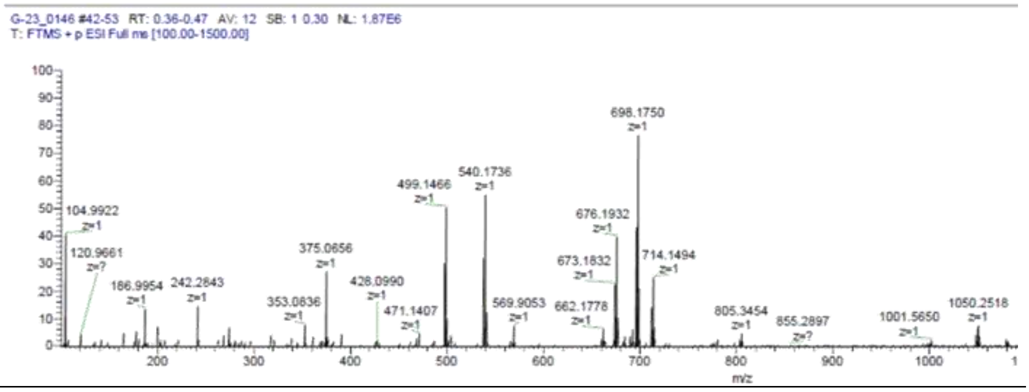

m/z (exp.)	m/z (calc.)	Species
676.1932	676.193	[Ir-C,$N_{Ph,Me}$ + Cys - Cl]$^+$
698.1750	698.175	[Ir-C,$N_{Ph,Me}$ + Cys + Na – H - Cl]$^+$
714.1494	714.149	[Ir-C,$N_{Ph,Me}$ + Cys + K – H - Cl]$^+$
499.1466	499.147	[Ir-C,$N_{Ph,Me}$ - Cl]$^+$

Figure S26. HRMS-ESI spectrum of the reaction of Ir-C,$N_{Ph,Me}$ and N-acetyl-L-cysteine methyl ester (Cys**)**.

The Ir-complex was dissolved in H_2O/MeOH 7:3 and reacted for 2 h at 37 °C with 2 eq. Cys ([**Ir-C,$N_{Ph,Me}$**] = 250 µM, [Cys] = 500 µM). The reaction mixture was diluted in MeOH for HRMS-ESI analysis. Peak assignment in the table.

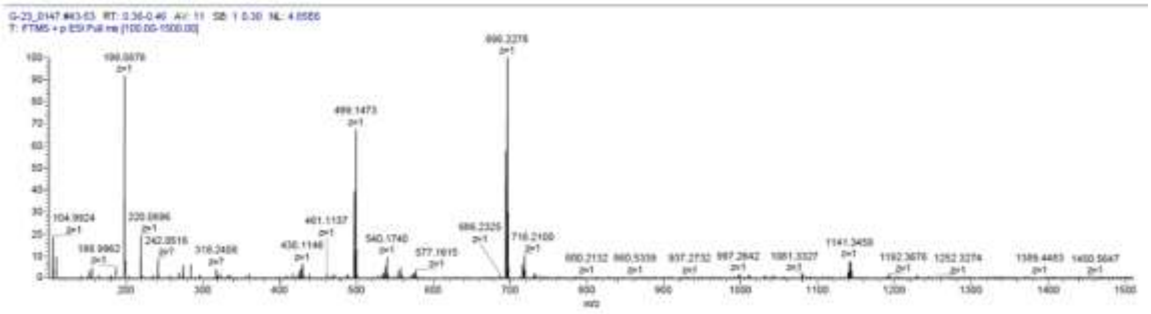

m/z (exp.)	m/z (calc.)	Species
696.2278	696.227	[Ir-C,$N_{Ph,Me}$ +His -Cl]$^+$
499.1473	499.147	[Ir-C,$N_{Ph,Me}$ - Cl]$^+$

Figure S27. HRMS-ESI spectra of the reaction of Ir-C,$N_{Ph,Me}$ and N-acetyl-L-histidine (His).

The Ir-complex was dissolved in H_2O/MeOH 7:3 and reacted for 2 h at 37 °C with 2 eq. His ([**Ir-C,$N_{Ph,Me}$**] = 250 µM, [His] = 500 µM). The reaction mixture was then diluted in MeOH for HRMS-ESI analysis. Peak assignment in the table.

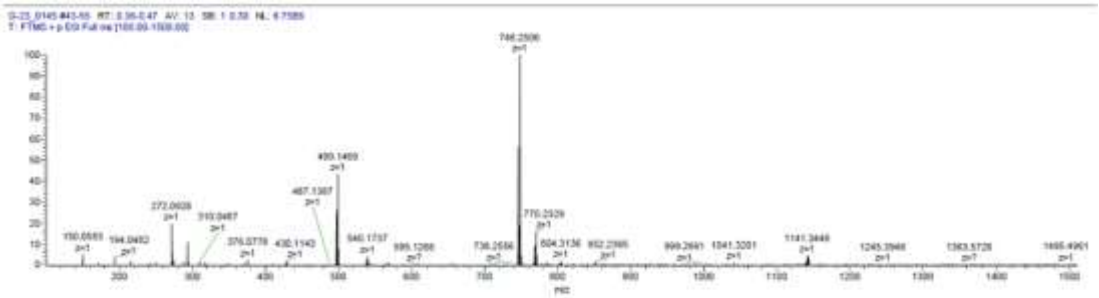

m/z (exp.)	m/z (calc.)	Species
748.2506	748.250	[Ir-C,$N_{Ph,Me}$ + Met -Cl]$^+$
770.2323	770.233	[Ir-C,$N_{Ph,Me}$ +Met + Na - H - Cl]$^+$
499.1473	499.147	[Ir-C,$N_{Ph,Me}$ - Cl]$^+$

Figure S28. HRMS-ESI spectrum of the reaction of Ir-C,$N_{Ph,Me}$ and N-Boc-L-methionine (Met).

The Ir-complex was dissolved in H_2O/MeOH 7:3 and reacted for 2 h at 37 °C with 2 eq. Met ([**Ir-C,$N_{Ph,Me}$**] = 250 µM, [Met] = 500 µM). The reaction mixture was then diluted in MeOH for HRMS-ESI analysis. Peak assignment in the table.

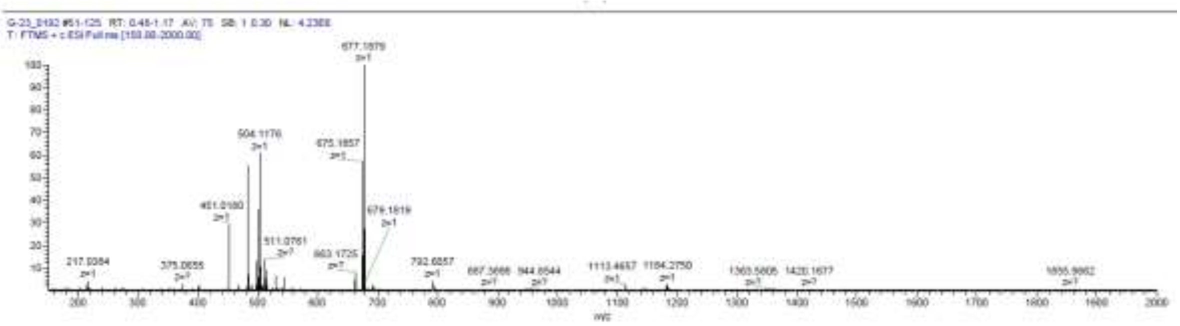

Figure S 2910. HRMS-ESI spectrum of the reaction between Ir-N,$N_{Py,Me}$ and N-acetyl-L-cysteine methyl ester (Cys).

The Ir-complex was dissolved in H_2O/MeOH 7:3 and reacted for 2 h at 37 °C with 2 eq. Cys ([**Ir-N,$N_{Py,Me}$**] = 250 µM, [Cys] = 500 µM). The reaction mixture was then diluted in MeOH for HRMS-ESI analysis. The peak at m/z 677.1879 (calcd. 677.1880) was assigned to [Ir-N,$N_{Py,Me}$ + Cys – H - Cl]$^+$ and identified as the adduct of the complex and Cys

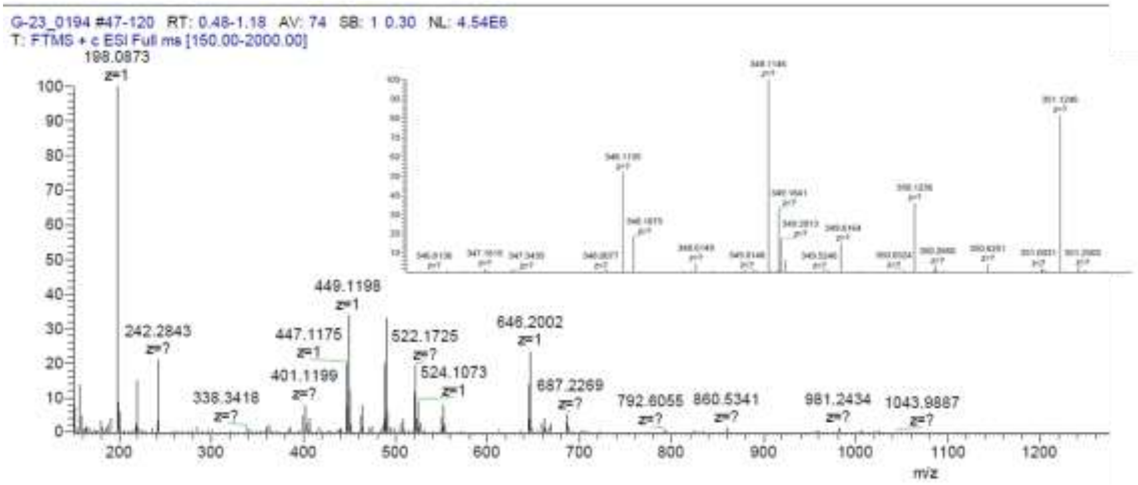

Figure S 30. HRMS-ESI spectrum of the reaction of Ir-N,N$_{Py,Me}$ and N-acetyl-L-histidine (His).

The Ir-complex was dissolved in H_2O/MeOH 7:3 and reacted for 2 h at 37 °C with 2 eq. His ([**Ir-N,N$_{Py,Me}$**] = 250 µM, [His] = 500 µM). The reaction mixture was then diluted in MeOH for HRMS-ESI analysis. The peak at m/z 349.1146 (calcd. 349.1149) was assigned to [Ir-N,N$_{Py,Me}$ + His - Cl]$^{2+}$ and identified as the adduct between the complex and His.

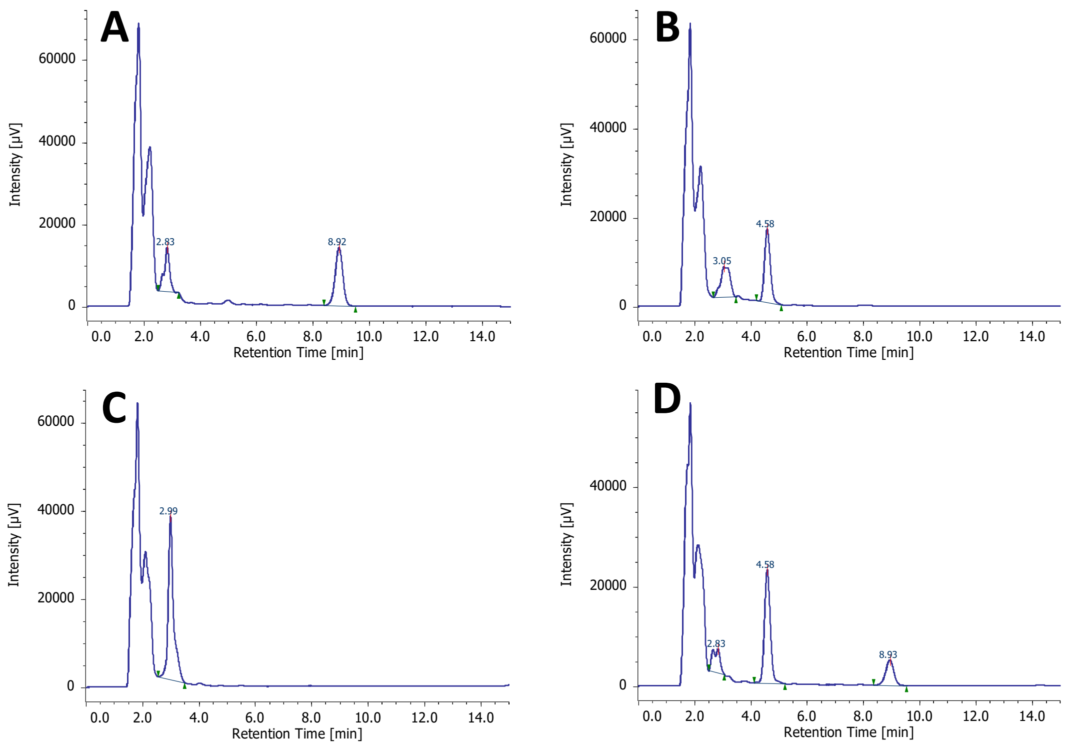

t_R (min)	assignment
8.9	Cys adduct
4.6	Met adduct
3.0	DMSO adduct
2.8	Unidentified species

Figure S 31. HPLC analysis of reaction mixtures of Ir-C,N$_{Ph,Me}$ with amino acid derivatives in DMEM/DMSO 99:1 (A-C) and competitive binding (D)

Ir-C,N$_{Ph,Me}$ (50 µM) was incubated in DMEM:DMSO 99/1 until complete conversion to the DMSO adduct. The following amino acids were added and the mixtures incubated at 37 °C for 2 h: (A) N-acetyl-L-cysteine methyl ester (**Cys,** 325 µM), (B) N-Boc-L-methionine (**Met**, 325 µM), (C) N-acetyl-L-histidine (**His**, 325 µM), (D) mixture of the 3 amino acid derivatives (325 µM each). In chromatogram C, no peaks attributable to Ir-His adduct were observed and as only the peak of starting DMSO-species is observed, suggesting that DMSO is not displaced by histidine. In chromatogram D, only the peaks assigned to Ir-Met and Ir-Cys adducts were observed (together with an unidentified species at 2.8 min), and the ratio of peak areas gives a [Ir-Met]/[Ir-Cys] ratio of 3:1, suggesting a preference for methionine over cysteine binding. HPLC conditions 1-3.

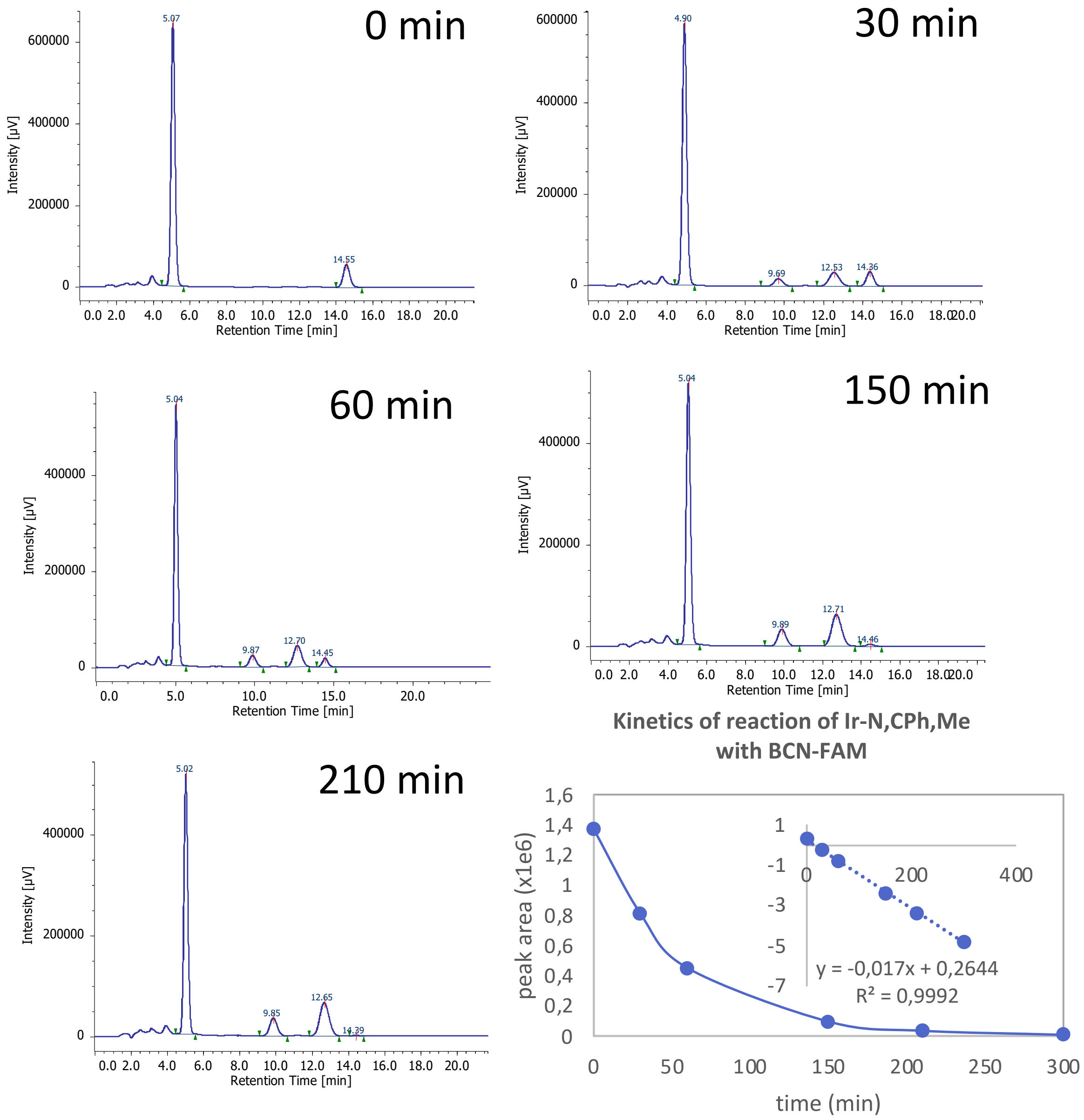

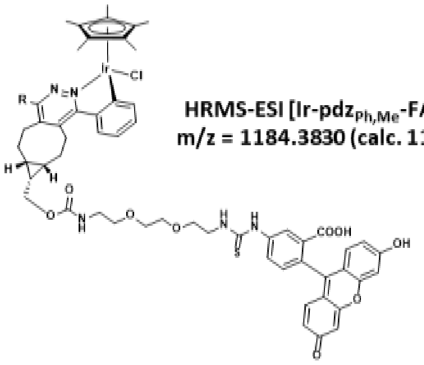

HRMS-ESI [Ir-pdz$_{Ph,Me}$-FAM – Cl]$^+$
m/z = 1184.3830 (calc. 1184.3819)

t_R (min)	assignment
5.1	**BCN-FAM**
14.5	**Ir-C,N$_{Ph,Me}$**
9.9 & 12.7	**Ir-pdz$_{Ph,Me}$-FAM** (pair of diastereomers)

Figure S 32. Chromatograms of the reaction mixture of Ir-C,N$_{Ph,Me}$ (50 µM) and BCN-FAM (125 µM) in MeOH recorded at different time points.

The kinetics is first order in complex with a rate constant k of 0.017 min^{-1} and a half-life of 41 min. The identity of the iEDDA product was assessed by HRMS-ESI analysis of the crude reaction mixture. HPLC conditions 1-3.

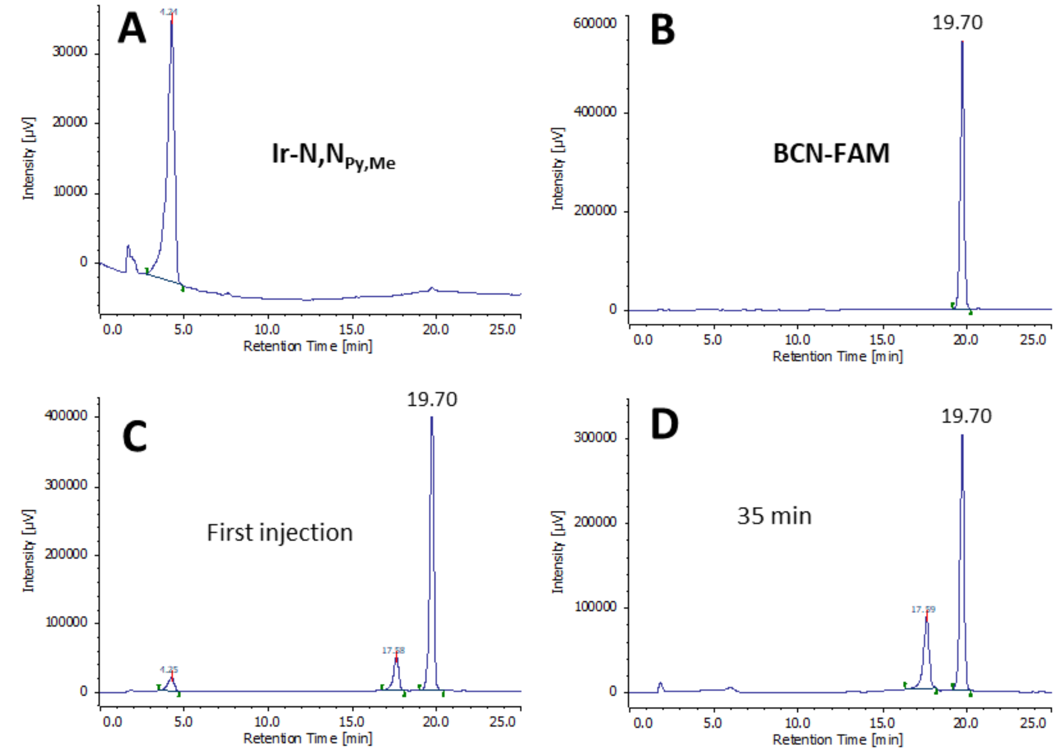

t_R (min)	assignment
19.7	**BCN-FAM**
4.3	**Ir-N,N$_{Py,Me}$**
17.6	**Ir-pdz$_{Py,Me}$-FAM**

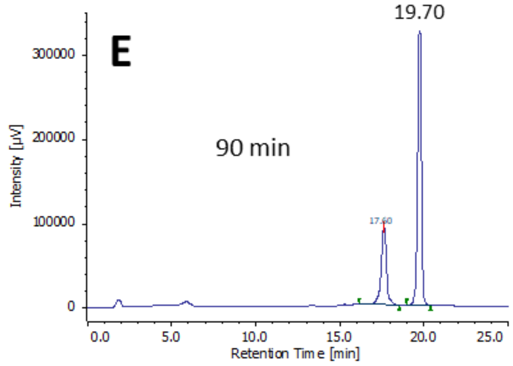

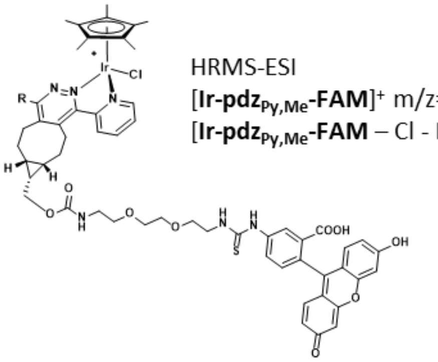

HRMS-ESI
[**Ir-pdz$_{Py,Me}$-FAM**]$^+$ m/z=1221.3547 (calc. 1221.3533)
[**Ir-pdz$_{Py,Me}$-FAM** – Cl - H]$^+$ m/z=1185.3790 (calc. 1185.37

Figure S 33. Chromatograms of Ir-N,N$_{Py,Me}$ (50 µM, A), BCN-FAM (125 µM, B) and reaction mixtures (C-E) in MeCN recorded at different time points

The identity of the iEDDA product was assessed by HRMS-ESI analysis of the crude reaction mixture. HPLC conditions 1-5.

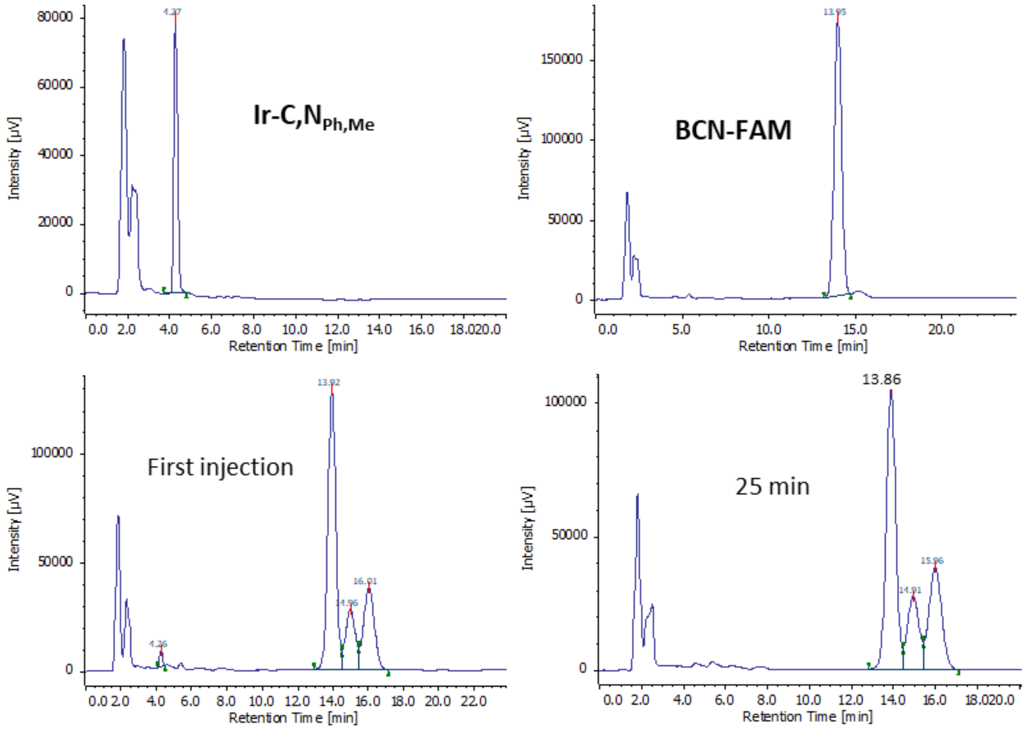

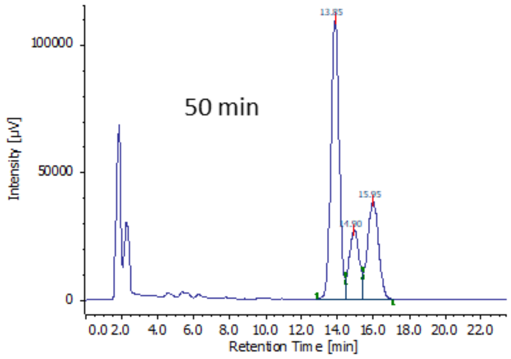

t_R (min)	assignment
14.0	**BCN-FAM**
4.3	**Ir-C,N$_{Ph,Me}$** (DMSO adduct)
14.9 & 16.0	**Ir-pdz$_{Ph,Me}$FAM** (pair of diastereomers

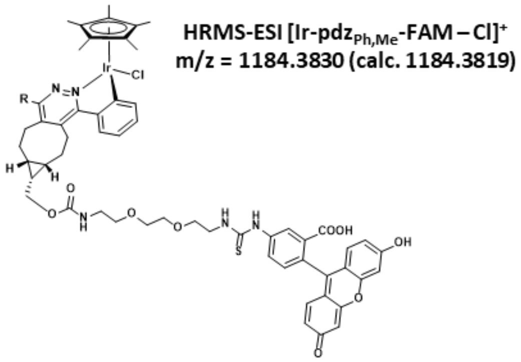

Figure S34. Chromatograms of Ir-C,N$_{Ph,Me}$ (50 µM), BCN-FAM (125 µM) and reaction mixtures in DMEM:DMSO 99/1 recorded at different time points. HPLC conditions 1-4.

The identity of the product was assessed by HRMS-ESI analysis of the crude reaction mixture. Of note, two peaks attributable to diastereomeric products were observed, derived from the combination of the enantiomerically pure BCN derivative with the two enantiomers of the Ir-complex which is chiral at the metal centre.

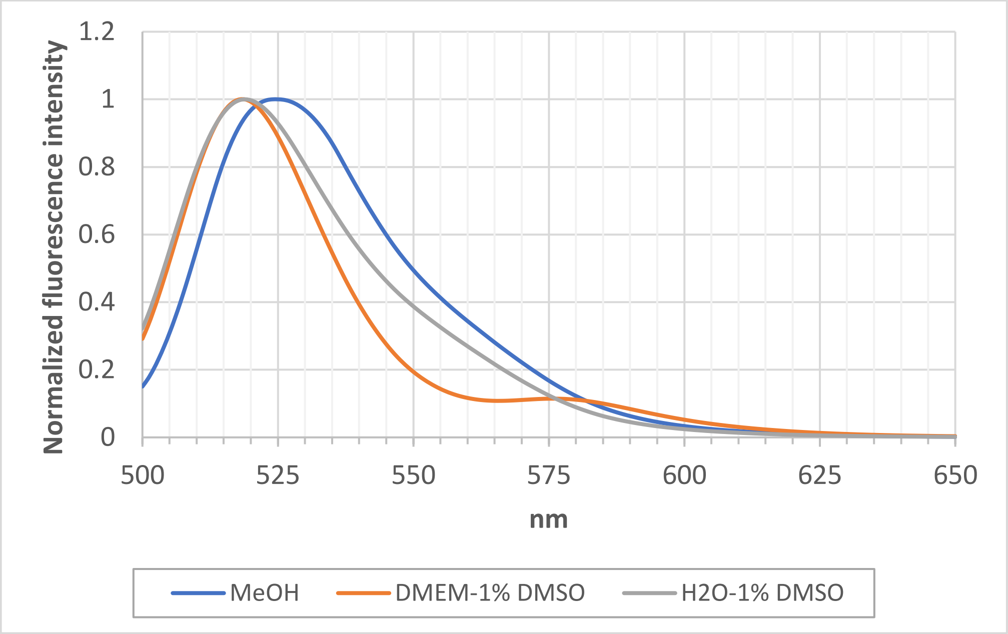

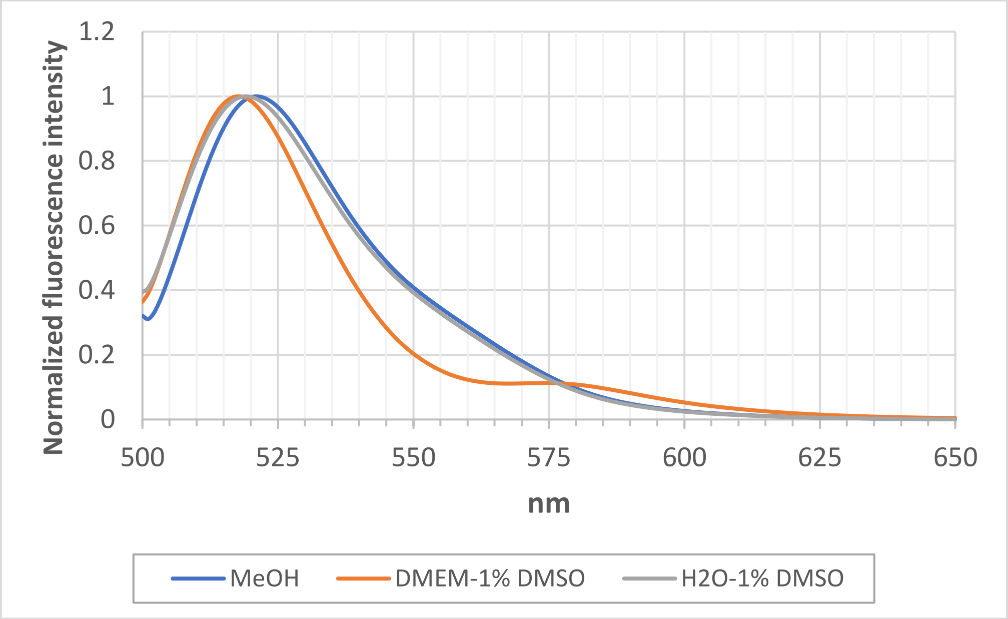

Figure S 35. **Fluorescence spectra of Ir-pdz$_{Ph,Me}$-FAM (top) and Ir-pdz$_{Py,Me}$-FAM (bottom).**

Ir-C,N,$_{Ph,Me}$ and **Ir-N,N,$_{Py,Me}$** were reacted with **BCN-FAM** in MeOH or MeCN ([Ir] = 150 µM, [BCN-FAM] = 50 µM) and monitored by HPLC until **BCN-FAM** was fully consumed. The reaction mixtures containing **Ir-pdz$_{R,R}$-FAM** were diluted to 5 µM in the different media and analyzed (λ_{ex} = 490 nm)

In-cell iEDDA post-labelling

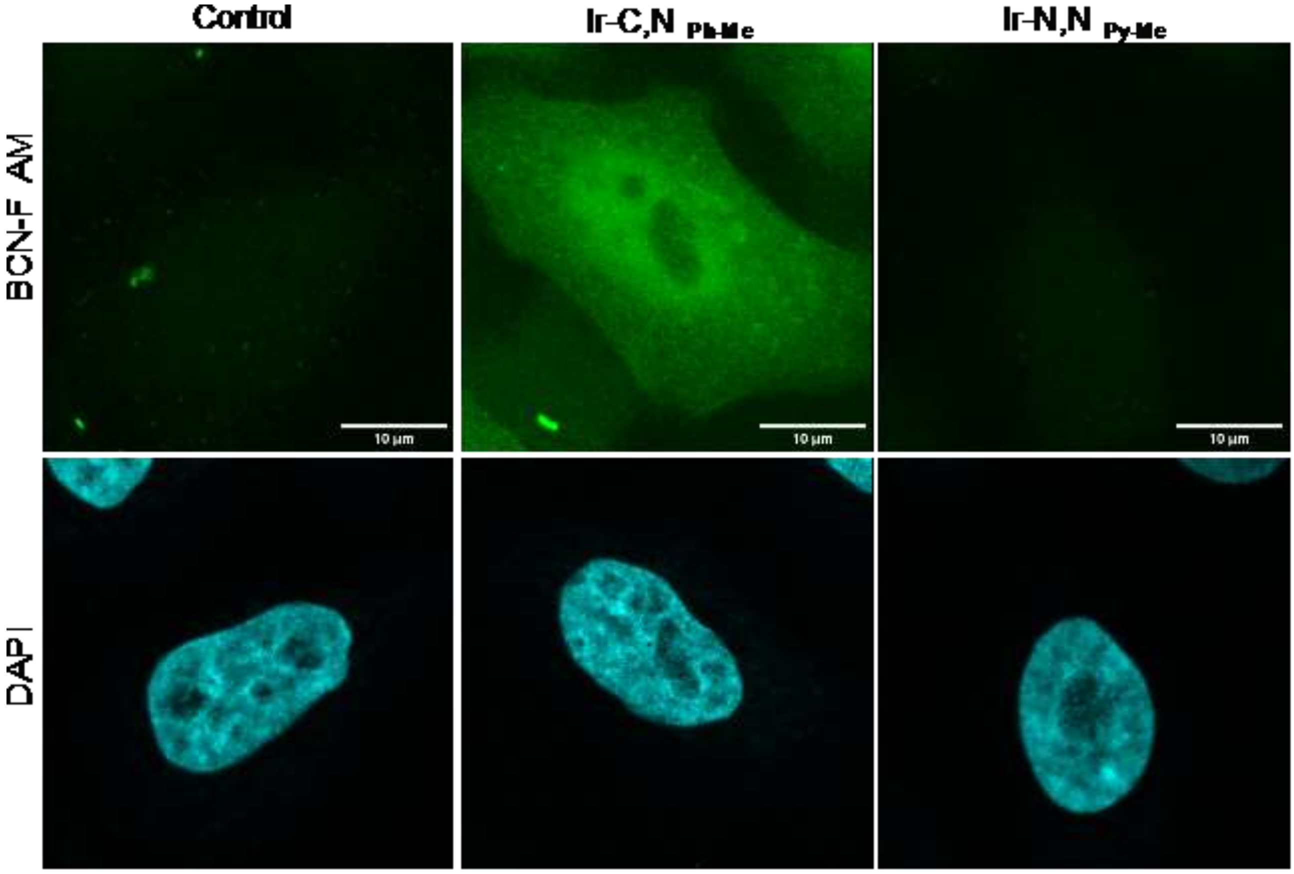

Fig S36. Confocal imaging of Ir-C,$N_{Ph,Me}$ in fixed HeLa cells using BCN-FAM as a sensor.

Live HeLa cells were exposed to 2 µM **Ir-C,$N_{Ph,Me}$** or **Ir-N,$N_{Py,Me}$** for 30 min, then fixed and stained with 8 µM BCN-FAM thanks to the iEDDA reaction. DNA was stained with DAPI. A robust fluorescent signal was detected in cells (nucleus and cytoplasm) exposed to **Ir-C,$N_{Ph,Me}$** but not in cells exposed to **Ir-N,$N_{Py,Me}$** or the DMSO vehicle, consistent with the results obtained in live cells. A less intense cytoplasmic labeling was also seen in these conditions.

Crystallographic Data and Tables

Table S 2. Crystallographic data for [Ir-N,$N_{Py,Me}$]PF_6, Ir-C,$N_{Py,Me}$, [Ir-N,$N_{Py,Py}$]PF_6 and [Ir_2(N,$N_{Py,Py}$)Cl_2]$(PF_6)_2$

	[Ir-N,$N_{Py,Me}$]PF_6	Ir-C,$N_{Py,Me}$	[Ir-N,$N_{Py,Py}$]PF_6	[Ir_2(N,$N_{Py,Py}$)Cl_2] $(PF_6)_2$
CCDC deposit number	*2324970*	*2324971*	*2324972*	*2324973*
Empirical formula[a]	$C_{18}H_{22}N_5F_6PClIr$	$C_{19}H_{22}N_4ClIr$	$C_{22}H_{23}ClF_6IrN_6P$	$C_{32}H_{38}Cl_2F_{12}Ir_2N_6P_2$
Moiety Formula	$C_{18}H_{22}Cl_1Ir_1N_5^+$, PF_6^-	$C_{19}H_{22}N_4ClIr$	$C_{22}H_{23}ClIrN_6^+$, PF_6^-	$C_{32}H_{38}Cl_2Ir_2N_6^{2+}$, 2 PF_6^-
Formula weight (g/mol)	681.02	534.05	744.08	1251.92
Temperature (K)	200	200	200	200
Crystal system	Triclinic	Monoclinic	Monoclinic	Triclinic
Space group	P-1	$P2_1/c$	P2/c	P-1
a (Å)	8.3924(2)	15.9414(4)	10.7994(7)	8.3698(4)
b (Å)	11.0045(3)	15.4460(4)	17.0415(10)	11.0136(5)
c (Å)	12.7830(3)	7.4467(2)	14.9040(8)	11.7126(6)
α (°)	77.956(2)	90	90	92.595(3)
β (°)	88.671(2)	91.931(2)	102.434(3)	110.256(3)
γ (°)	76.559(2)	90	90	104.613(3)
Volume (Å^3)	1122.55(5)	1832.57(8)	2678.6(3)	969.63(8)
Z	2	4	4	1
ρ_{calc} (g/cm^3)	2.015	1.936	1.845	2.144
Absorption coefficient μ (mm^{-1})	13.898 (CuKα)	15.509 (CuKα)	11.728 (CuKα)	15.981 (CuKα)
F(000)	656	1032	1440	598
Crystal size (mm²)	0.30 × 0.20 × 0.20	0.54 × 0.07 × 0.03	0.20 × 0.10 × 0.06	0.39 × 0.13 × 0.08
Wavelength λ (Å)	1.54178	1.54178	1.54178	1.54178
2Θ range (°)	8.448 - 133.16	7.972 - 133.232	7.988 - 133.244	10.802 - 133.45
Miller indexes ranges	-9 ≤ h ≤ 9, -13 ≤ k ≤ 9, -15 ≤ l ≤ 15	-18 ≤ h ≤ 18, -18 ≤ k ≤ 18, -8 ≤ l ≤ 8	-12 ≤ h ≤ 12, -20 ≤ k ≤ 20, -17 ≤ l ≤ 12	-8 ≤ h ≤ 9, -13 ≤ k ≤ 13, -13 ≤ l ≤ 13,
Measured reflections	13460	15389	23660	13937
Unique reflections	3944	15389	4718	3407
R_{int} / R_{sigma}	0.0437 / 0.0394	0.0430 / 0.0583	0.0685 / 0.0503	0.0519 / 0.0435
Reflections [I ≥ 2σ(I)]	3655	10983	3769	3063
Restraints	0	0	0	36
Parameters	295	227	341	258
Goodness-of-fit F²	1.055	0.987	1.010	1.079
Final R indexes[b c] [all data]	R1 = 0.0300, wR2 = 0.0660	R1 = 0.0575, wR2 = 0.1160	R1 = 0.0447, wR2 = 0.0758	R1 = 0.0422, wR2 = 0.0905
Final R indexes[b c] [I ≥ 2σ(I)]	R1 = 0.0273, wR2 = 0.0649	R1 = 0.0408, wR2 = 0.1048	R1 = 0.0314, wR2 = 0.0700	R1 = 0.0362, wR2 = 0.0868
Largest diff. peak/hole (e/Å^3)	0.61 / -1.42	0.90 / -1.64	0.81 / -0.58	1.88 / -0.94

[a] Including solvent molecules (if presence); [b] $R1 = \sum\left||F_o| - |F_c|\right| / \sum |F_o|$; [c] $wR2 = \sqrt{\sum\left(w(F_o^2 - F_c^2)\right)/\sum\left(w(F_o^2)^2\right)}$

Table S 3. Selected bond distances (Â) for Ir-N,$N_{Py,Me}$, Ir-N,$N_{Py,Py}$ and Ir-C,$N_{Ph,Me}$

Compound	Ir-centroid	Ir-Cl	Ir-N(tz)	Ir-N(py)	Ir-C8
**Ir-N,$N_{Py,Me}$**	1.793	2.385(1)	2.058(4)	2.107(5)	-
**Ir-N,$N_{Py,Py}$**	1.801	2.386(1)	2.073(5)	2.110(3)	-
**Ir-C,$N_{Ph,Me}$**	1.831	2.379(2)	2.024(7)	-	2.049(9)

Table S 4. Selected bond angles (°) for Ir-N,$N_{Py,Me}$, Ir-N,$N_{Py,Py}$ and Ir-C,$N_{Ph,Me}$

Compound	N(tz)-Ir-Cl	N(py)-Ir-Cl	N(tz)-Ir-N(py)	C-Ir-Cl	C-Ir-N(py)
**Ir-N,$N_{Py,Me}$**	87.13	82.63	78.45	-	-
**Ir-N,$N_{Py,Py}$**	86.34	83.63	78.59		
**Ir-C,$N_{Ph,Me}$**	78.59	-	-	84.70	78.59

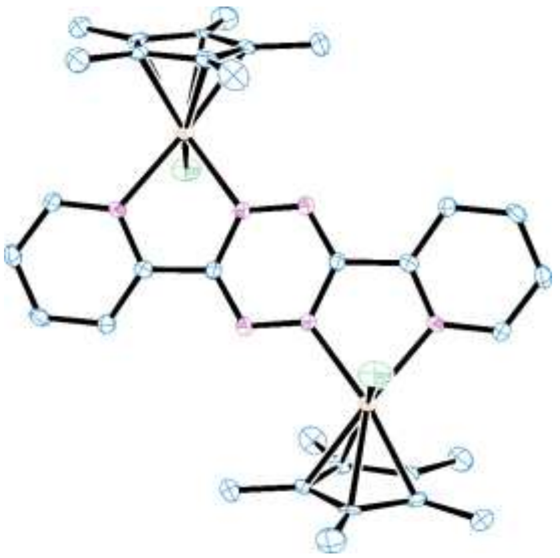

Figure S 37. X-ray crystal structure of $[Ir_2(N,N_{Py,Py})Cl_2](PF_6)_2$.

X-ray crystallographic structure of the dinuclear complex obtained as a side product during the synthesis of **Ir-N,$N_{Py,Py}$** with thermal ellipsoids drawn at 30% probability. Hydrogen atoms and PF_6 counterions have been omitted for clarity
